# Evaluating the administration costs of biologic drugs: development of a cost algorithm

**DOI:** 10.1186/s13561-014-0026-2

**Published:** 2014-10-23

**Authors:** Ebenezer K Tetteh, Stephen Morris

**Affiliations:** Department of Applied Health Research, EPSRC Centre for Innovative Manufacturing in Emergent Macromolecular Therapies, University College London, 1-19 Torrington Place, London, WC1E 7HB UK

**Keywords:** Administration costs, Biologics, Economic evaluation, Formulation, Manufacturing

## Abstract

Biologic drugs, as with all other medical technologies, are subject to a number of regulatory, marketing, reimbursement (financing) and other demand-restricting hurdles applied by healthcare payers. One example is the routine use of cost-effectiveness analyses or health technology assessments to determine which medical technologies offer value-for-money. The manner in which these assessments are conducted suggests that, holding all else equal, the economic value of biologic drugs may be determined by how much is spent on administering these drugs or trade-offs between drug acquisition and administration costs. Yet, on the supply-side, it seems very little attention is given to how manufacturing and formulation choices affect healthcare delivery costs. This paper evaluates variations in the administration costs of biologic drugs, taking care to ensure consistent inclusion of all relevant cost resources. From this, it develops a regression-based algorithm with which manufacturers could possibly predict, during process development, how their manufacturing and formulation choices may impact on the healthcare delivery costs of their products.

**Electronic supplementary material:**

The online version of this article (doi:10.1186/s13561-014-0026-2) contains supplementary material, which is available to authorized users.

## Background

The adoption and utilization of beneficial medical technologies including biologic drugs has, in recent times, been subject to a number of regulatory, marketing, reimbursement (coverage) and other demand-side hurdles including the so-called risk-sharing arrangements. These demand-side hurdles have evolved out of increasing healthcare payer concerns about the high acquisition costs and budgetary impacts of these medical technologies. Notably, healthcare payers have turned to the use of cost-effectiveness analysis (CEA), budget impact analysis or health technology assessments (HTA) to estimate cost-effectiveness and affordability prior to deciding whether to adopt or reimburse the utilization of biologics within their original or restricted marketing authorization or not. This typically involves comparing, over a specified time period, total healthcare delivery costs (i.e., the sum of drug acquisition costs plus administration and future healthcare-related costs) associated with a given technology and the total health benefits expected. Holding all else constant, the estimated cost-effectiveness of a biologic drug may be dictated by how much of healthcare resources are spent on drug administration, and whether trade-offs exist between drug acquisition and administration costs.

On the supply-side, it has been observed that biological drug candidates are developed with a skewed focus on clinical efficacy and safety to the neglect of issues related to the ease of manufacturing, affordability and cost-effectiveness of these therapies to healthcare payers. This often leads to unnecessary waste and excessive reworking of manufactured products, and a growing concern over the failures and struggles manufacturers face in passing through what is becoming an increasingly complex set of regulatory and demand-restricting hurdles. That latter is known to be associated with significant delays in market launch, in addition to the time and revenue lost in price negotiations [1]. So besides worrying about the ease of manufacturing, it is also useful for manufacturers to, at least, consider prior to market launch, the administration and total healthcare delivery costs associated with the different ways they choose to manufacture and formulate their products. In that case, manufacturers' evaluation of drug administration costs should be done in the same manner as it will be conducted by healthcare payers. The aim of this study is to evaluate the administration costs of biologic drugs, to identify the factors that affect variation in these costs, and argue why such evaluations are an important step in biopharmaceutical manufacturing. That is, we explore why pharmaceutical manufacturers should consider the link between administration costs (and how this is influenced by formulation and manufacturing), total healthcare delivery costs and value-for-money when making their go-no-go R&D decisions.

Our study objectives and design are motivated by two key points. First, in a systematic review of the economic value of reducing medication dosing frequency using drug delivery systems, Cheng et al. [2] found that, in most cases, drug products with less-frequent dosing schedules tend to be cost effective when compared to conventional (standard) formulations containing the same active moiety – although these `advanced' or `improved' delivery systems may be expensive to make. Second, a recent systematic review of studies reporting on the costs of administering biologics within the United Kingdom (UK) National Health Service (NHS) identified possible trade-offs between acquisition and administration costs: a drug that appears cheap to buy may (in the long-run) have higher total healthcare delivery costs as more NHS resources are spent on drug administration. The budgetary impact of a biologic with high acquisition costs but relatively low administration costs could be the same as or lower than that of a less expensive biologic with higher administration costs. The study also found that there are inconsistencies in how studies define administration costs and consequently, differences in the type of costs included or excluded from estimates of drug administration costs[3]. These differences in what cost items are included or excluded means some of the reported differentials in administration costs for biologic drugs may not be real and cannot be used unreservedly in economic analyses. Once differences in cost estimates simply reflect differences in methods of measurement, one cannot tell for sure whether trade-offs exist between administration costs and drug acquisition costs or not; and to what extent administration costs could influence conclusions reached about cost-effectiveness.

Taking into account these points, we evaluate variations in the administration costs for a sample of eighteen biologic drugs listed for use in the UK NHS, taking care to ensure consistent inclusion or exclusion of all relevant costs related to drug administration. We do this to ensure very little variation in drug administration costs can be attributed to differences in the method of measurement. We develop an administration-cost algorithm to help manufacturers predict, prior to market launch, the administration costs associated with their formulation choice for each biologic drug candidate in their portfolio. We believe this, together with manufacturers' expectations of product prices, should help them consider the possible trade-offs between drug acquisition and administration costs; and generate credible estimates of total healthcare delivery costs of their drug products and the likelihood that these products will receive favourable recommendations from healthcare payers.

The paper is structured as follows. We first describe in Section Methods our methodological approach, underlying assumptions made in our analyses, and the data sources used. This is followed by Sections Results and Discussion with our results and discussion points. Section Conclusions completes the paper with our conclusions.

## Methods

To avoid overcomplicating our analyses, we will assume *clinical outcome neutrality*; that is, for any comparison of different modes of administering a biologic drug, there are no differences in net health benefits (i.e., efficacy minus safety concerns) or that differences in net health benefits have no bearing on the magnitude or variation in administration costs. For example, differences in the incidence and severity of adverse events between two or more formulations of a given biologic drug will have no bearing as to how much is spent on drug administration costs. We also ignore other costs associated with disease management.

### Identifying and measuring administration costs

**Figure 1:**
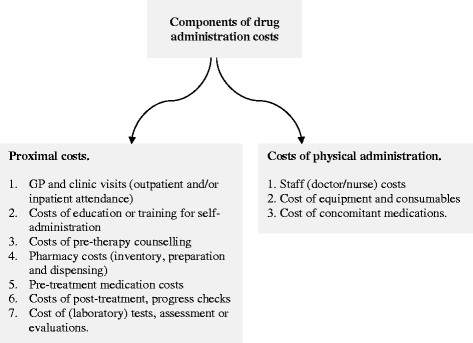
**Framework of drug administration costs.**

From an economic perspective, costs measured should reflect the opportunity costs of NHS resources deployed in administering biologic drugs that could otherwise have been used elsewhere had the drug in question not been administered. An accurate measurement of drug administration costs thus requires identifying *all* resources that will be expended or the `cost centres' where resources will be consumed and costs incurred [4]. To identify the `cost centres' related to the administration of biologic drugs (from a healthcare payer perspective), we employ the framework described by Tetteh and Morris [3] that makes a distinction between the proximal costs of drug administration and the costs of physical administration. In that framework (see Figure 1), proximal administration costs (Pc) refer to costs incurred before or after physical administration of the drug into the body whilst physical administration costs (PAc) refer to the costs of physically introducing the drug into a patient via one of the established routes for administration. Each component labelled in that framework constitutes a (micro-level) cost centre where resources are consumed and costs incurred.

We use this framework to ensure consistency in what type of administration costs are included or excluded in the analysis. Using a common yardstick should support (1) complete or near complete accounting of the opportunity costs associated with drug administration, and (2) "apples to apples" comparison of the administration costs of biologic drugs such that very little variation in administration costs can be attributed to the method of measurement. For the same reasons, we defined a common time frame over which drug administration costs will be estimated. We chose to evaluate annual costs of drug administration costs as this fitted well with the dosing regimens of all products in our sample. For simplicity, we base our analysis on a single patient who successfully completes a single full treatment course over a 12 month period. It might be argued that this will introduce bias against biologics indicated for an acute illness with typically `short' treatment episodes. However, extending the time frame beyond one-year period will actually amplify the cost differences between acute and chronic biologics whilst a 6-month period will not fit with the dosing regimen of some of the products in our sample. What is more, we do not consider repeat treatment episodes over the one-year period. Hence, our estimates of annual drug administration costs should not be biased against biologics indicated for acute illnesses.

### Product sample and dosing regimens modelled

Our analysis makes use of an unbalanced sample of 18 therapeutically-active biologics; of which eight are administered intravenously, eight given by subcutaneous delivery and two given intramuscularly. Within this sample, fifteen of the products are humanized monoclonal antibodies (mAb) with the remainder comprising of one fragmented monoclonal antibody (fAb), a fusion protein and an interferon protein. The characteristics of this product sample are presented in Table 1. We make no argument that this sample is representative of all existing or emergent biologics or macromolecular therapies.

**Table 1:** **Characteristics of product sample**

Product name	Product type	Clinical indication considered	Disease type
**Biologics given intravenously**			
Basiliximab^†^, Simulect®	mAb	Prophylaxis of acute organ rejection in allogeneic renal transplantation.	Acute
Bevacizumab, Avastin®	mAb	First-line treatment of adult patients with non-small cell lung cancer.	Chronic
Cetuximab, Erbitux®	mAb	Treatment of epidermal-growth-factor-receptor (EGFR)-expressing, Kirsten Rat Sarcoma-2 (KRAS) wild-type metastatic colorectal cancer.	Chronic
Infliximab, Remicade®	mAb	Treatment of active rheumatoid arthritis that is unresponsive to disease modifying anti-rheumatic drugs (DMARDs) or those with severe active disease not previously treated with methotrexate (MTX) or DMARDs.	Chronic
Oftamumab^†,^, Arzerra®	mAb	Treatment of chronic lymphocytic leukaemia that is refractory to fludarabine and alemtuzumab.	Chronic
Panitumumab, Vectibix®	mAb	Treatment of wild-type KRAS metastatic colorectal cancer.	Chronic
Tocilizumab, Actemra®	mAb	Treatment of moderate-to-severe active rheumatoid arthritis.	Chronic
Trastuzumab, Herceptin®	mAb	Treatment of advanced and metastatic breast cancer.	Chronic
**Biologics given subcutaneously**			
Adalimumab^†^, Humira®	mAb	Treatment of moderate-to-severely active Crohn's disease in children.	Chronic
Canakinumab^†^, Ilaris®	mAb	Treatment of cryopyrin-associated periodic syndromes (CAPS) in adults.	Chronic
Certolizumab pegol^†^, Cimzia®	fAb	Treatment of moderate-to-severe active rheumatoid arthritis in adults when response to DMARDs including MTX has been inadequate.	Chronic
Denosumab^†^, Prolia®	mAb	Treatment of osteoporosis in post-menopausal women at increased risk of fractures.	Chronic
Etanercept^†^, Enbrel®	Fusion protein	Treatment of active and progressive psoriatic arthritis that has not responded adequately to DMARDs.	Chronic
Golimumab^†^, Simponi®	mAb	Treatment of moderate-to-severe active ankylosing spondylitis.	Chronic
Omalizumab^†^, Xolair®	mAb	Management of immunoglobulin-E mediated asthma.	Chronic
Ustekinumab^†^, Stelara®	mAb	Treatment of moderate-to-severe plaque psoriasis.	Chronic
**Biologics given intramuscularly**			
Palivizumab^†^, Synagis®	mAb	Prevention of serious lower respiratory tract disease caused by respiratory syncytial virus (RSV) in children at high risk of RSV disease.	Acute
Interferon beta-1a^†^, Avonex®	Interferon protein	Treatment of relapsing multiple sclerosis in adult patients, i.e., two or more acute exacerbations in the previous three years without evidence of progressive disease.	Chronic

Notes: ^†^refers to biologics that are sold bundled with some of the equipment and consumables used in drug administration.

To estimate the costs of administering the biologic drugs in our sample, some idea or knowledge of the dosing regimen for patients considered eligible to receive a given biologic drug is needed. We follow the dosing regimen indicated by the marketing authorisation for a given biologic drug, gathering this information from the posology described in the drug's package inserts, the summary of product characteristics (S*m* PC) posted on the European Medicines Agency (EMA) website; the British National Formulary (BNF) or the electronic Medicines Compendium (eMC). Obviously, within UK NHS settings, the prescribed pathway suggested by the regulatory license or marketing authorization may not necessarily coincide with actual clinical practice – bearing in mind possible gaps between recommendations in HTA guidance or clinical guidelines and implementation of these recommendations in routine practice; as well as practice-specific watch-and-wait treatment strategies. To avoid the complexity introduced by what happens in routine clinical practice, we simply modelled the dosing instructions given in the products' package inserts or the S*m* PC. For this, we assumed continuous dosing of a given biologic for the whole year unless the marketing authorization or S*m* PC clearly states the maximum number of doses or recommended duration of treatment. This is because we found it hard to make any unquestionable assumption about the proportion of treatment-responders and non-responders. The dosing regimens modelled will be found in Additional file 1: Appendix A.

### Analysis

We first conducted a deterministic analysis with the estimated costs of drug administration disaggregated into proximal costs and the cost of physically administering the drug. We used Figure 1 as a guide to selecting which cost item to include as long as we found publicly-available data for that cost item. Our analysis was done using a spreadsheet model with data inputs from the BNF and eMC, the NHS Reference Costs 2011-2012, the NHS electronic Drug Tariff and the 2012 edition of the PSSRU (Personal Social Services Research Unit) Costs for Health and Social Care, and from published and grey literature. A summary of the data inputs will be found in Additional file 2: Appendix B. One issue with our deterministic analysis is the well-known fact that there is: (1) uncertainty in the incidence and severity of illness and for that matter, demands for health intervention using biologic drugs plus (2) uncertainty with regards to treatment outcomes, which fuels future demands for healthcare intervention; for example, dose reduction or escalation; modifications to dosing regimens and treatment protocols and/or deployment of alternative (salvage) interventions in complementary or substitutive ways [5],[6]. For this reason, the quantity and costs of NHS resources expended on the administration of biologic drugs cannot be described by fixed values – considering also deviations of what happens in routine clinical practice from the EMA-approved posology.

We attempt to resolve this issue by introducing parameter uncertainty into our analysis – by fitting a gamma distribution to the deterministic estimates of Pc as well as PAc, and running 1000 Monte Carlo simulations^a^ for each product. Note that the choice of a gamma distribution is not arbitrary but reflects the observation that healthcare resource use and costs are skewed with non-negative values ranging from zero to positive infinity. As argued by Nixon & Thompson [7], skewed parametric (gamma, log-logistic, lognormal) distributions fit medical cost data better than a normal distribution and should in principle be preferred for estimation. What is not clear, however, is which skewed parametric distribution is best. Here we chose a two-parameter (α, β) gamma distribution, and given the absence of real-life data that reflects the uncertainty of healthcare demands requiring intervention with biologics, we adopted the simplest assumption that the mean and SE for administration costs for any given biologic drug in our sample is the same (see Briggs et al. [8]). That is, we apply a gamma distribution defined by α = 1 and β = ADMINCOST. We do not expect this to introduce any systematic bias as the assumption will apply to all products within our sample.

From the synthetic dataset generated, we estimated the proportion of total administration costs that is due to Pc or PAc for our sample of biologic drugs (categorized according to their respective routes of administration) by graphing a log-log plot of Pc versus PAc. We took logs of Pc and PAc because of the skewness of a gamma-distributed cost data and to narrow down the range of (large) values. We then estimated the proportion of simulations where the ratio of proximal costs to physical administration costs (PAc/Pc) is greater than or equal to 1. The essence of this exercise is to identify the source of administration cost savings from changes in drug formulation and manufacturing.

### Identifying the algorithm

Recall that one of our study objectives is to develop an algorithm that will allow manufacturers to predict how administration costs change with how they choose to manufacture and formulate a given biologic drug candidate. On *a priori* grounds, we defined this algorithm as a sample regression function linking drug administration costs (ADMINCOST) and a number of independent explanatory variables (*X*). The explanatory variables we chose are those that we believe (bio)pharmaceutical manufacturers will have some information on, or an idea of, as they work on a number of promising biologic drug candidates, and decide which candidates to take forward to the next stage of process R&D or product development, and which ones to reserve as contingency or backup options.

Given the expected skewness of our simulated data for ADMINCOST (on the raw scale), we estimate the following with a log-transformed dependent variable:

$$\ln\left(\mathrm{ADMINCOST}\right)=\;\alpha +\beta_{0}\mathrm{SUBCUTANEOUS}+\beta_{1}\mathrm{INTRAMUSCULAR}+\beta_{2}\mathrm{DOSFREQ}+\beta_{3}\mathrm{PRODUCTBUND}+\beta_{4}\mathrm{INDICATN}+\beta_{5}\mathrm{DOSFRE}Q^{2}+\beta_{6}\mathrm{DOSFREQ}.\mathrm{INDICATN}+∊$$

The intercept is *α* and the error term (∊) represents any unexplained variation in administration costs. SUBCUTANEOUS is a dummy variable that takes the value of one if a product is given subcutaneously and zero otherwise. Equally, the dummy variable INTRAMUSCULAR takes on the value of one if a product is administered intramuscularly and zero otherwise. Intravenous administration is therefore the baseline, benchmark or reference category. We do this because for most biologics, intravenous infusion is the default (conventional) drug delivery or formulation choice given the fragility and poor oral bioavailability of macromolecular proteins. The variable DOSFREQ refers to the frequency (intensity) of dosing, which we define as the number of `unit administrations' in a given year. PRODUCTBUND is a zero-one dummy variable indicating whether a given biologic product is sold together with some of the equipment and consumables used in drug administration (1) or not (0). Similarly, INDICATN is a zero-one dummy variable indicating whether a biologic drug is for the management of an acute illness (0) or for a chronic illness (1). The interaction term DOSFREQ.INDICATN is intended to capture the notion that treatments for acute illnesses tend to have less `complex' dosing regimens compared to those for chronic illnesses; and the quadratic term DOSFREQ^2^ is intended to determine whether the marginal effects of DOSFREQ are increasing or diminishing as the frequency (intensity) of dosing increases.

We estimate the four nested models using *linear* ordinary least squares (OLS) regression. We labelled the four nested models as A, B, C and D – all of which rely on different combinations of the explanatory variables defined above. We designate C as the full unrestricted model as it includes all the explanatory variables above.

### Robustness checks

A well-known problem with OLS regression using a log-transformed dependent variable is that retransformation of ln $\mathrm{ADM}\hat{\mathrm{IN}}\mathrm{COST}$ to the raw untransformed scale gives the geometric mean for $\mathrm{ADM}\hat{\mathrm{IN}}\mathrm{COST}$ (which is often close to the median) rather than the arithmetic mean, the parameter of interest. (The hat on ADMINCOST indicates an estimated or predicted value). The problem is resolved by using what is called a smearing factor to minimize the prediction error. The name is derived from the fact that the factor distributes (smears) the `excess' or prediction error in one observation to other observations proportionally when adjusting unlogged median estimates to unlogged mean estimates. Generally, this takes the form: $\mathrm{ADM}\hat{\mathrm{IN}}\mathrm{COST}\;=\;\exp\left(X\hat{\beta_{k}}\right)\hat{•\phi }$ , where $\hat{\phi }$ is the smearing factor. We looked at three ways of deriving less-biased smearing estimates of ADMINCOST (on the raw untransformed scale).

The first method yields what is called `normal theory estimates' that are derived under the assumption that the error term for ln $\mathrm{ADM}\hat{\mathrm{IN}}\mathrm{COST}$ is normally distributed, in which case the smearing estimate of $\mathrm{ADMINCOST}=\exp\left(X{\hat{\beta }}_{k}+0.5{\hat{\sigma }}^{2}\right)$ where ${\hat{\sigma }}^{2}$ is the square of SE(ln $\mathrm{ADM}\hat{\mathrm{IN}}\mathrm{COST}$) and the smearing factor $\hat{ф}=\exp\left(0.5{\hat{\sigma }}^{2}\right)$. If errors are not normally distributed but are homoscedastic (i.e., constant variance), then the second method, which uses non-parametric (sub-group specific) smearing factors can be used to minimize prediction errors. The non-parametric smearing factor is given by: ф^=N-1∑i=1Nexp∊i^ , where N is the number of observations and $\hat{{∊}_{i}}$ is the log-scale residuals from the regression. When the errors are heteroskedastic (i.e., non-constant variance), or they depend on the explanatory variables, it is "better" to use a subgroup-specific smearing factor for each intravenous, subcutaneous or intramuscular product category [9],[10],[11]. The third method uses a regression-through-the-origin approach suggested by Wooldridge [12]. This is as follows: obtain for each observation the naϊve estimates $\hat{m_{i}}=\exp(\mathrm{ADM}\hat{\mathrm{IN}}\mathrm{COST})$; then perform a regression of ADMINCOST on $\hat{m_{i}}$ through the origin and obtain the only coefficient $\hat{\alpha_{0}}$ as the smearing factor.

Irrespective of the smearing factor used, its value in minimizing prediction error depends crucially on the presence and nature of heteroskedasticity in the log scale residuals. For example, the subgroup smearing factors assume that log scale heteroskedasticity varies across the mutually-exclusive subgroups specified. If, however, heteroskedasticity varies according to one or more (continuous, discrete or interacted) explanatory variables in the log-OLS regression, then we will have smearing estimates of ADMINCOST that are still biased. It is possible to run an auxiliary regression of the heteroskedastic variance as a function of one or more of the explanatory variables, i.e., $\hat{ф}=\exp(\hat{{∊}_{i})}=\rho \left(X\right)$; where ρ is a vector of regression coefficients. This crucially depends on how much of the heteroskedastic variance is explained by the chosen set of explanatory variables. Generally, this auxiliary-regression approach is thought to be cumbersome and there is no simple fix if the form of heteroskedasticity is unknown. An alternative estimator, however, exists in the form of generalized linear modelling (GLM) to overcome the retransformation problem.

GLM does this by directly and independently specifying: (1) a link function between the raw scale ADMINCOST and the linear index (*Xβ*_*k*_), and (2) a family of parametric distributions to reflect any heteroskedastic relationship between the raw scale error variance and $\mathrm{ADM}\hat{\mathrm{IN}}\mathrm{COST}$; i.e., var $\left(\mathrm{ADMINCOST}\right)≌\;\varphi .{\left[\mathrm{ADM},\hat{\mathrm{IN}},\mathrm{COST}\right]}^{\delta }$, where δ is the over-dispersion parameter and φ is a constant [11],[13],[14]. Independent specification of the link function (i.e., the scale of estimation) and family distributions (i.e., the variance function) under GLM allows $\mathrm{ADM}\hat{\mathrm{IN}}\mathrm{COST}$ to be estimated directly (or from the natural exponent of ln $\mathrm{ADM}\hat{\mathrm{IN}}\mathrm{COST})\;$ without the need for smearing factors. In most applications, three types of link functions are specified: "identity", "log" and "power". Here an identity link specifies the following relationship: $\mathrm{ADM}\hat{\mathrm{IN}}\mathrm{COST}=X\beta_{k}$; and the log links takes the form $\mathrm{ADM}\hat{\mathrm{IN}}\mathrm{COST}=\exp\left(X\beta_{k}\right)$. The family distributions commonly investigated or used to model heteroskedasticity are Gaussian if the parameter *δ* = 0; Poisson if *δ* = 1, Gamma or heteroskedastic normal if *δ* = 2 and inverse Gaussian if *δ* = 3. The appropriate family distribution is often identified using the so-called modified Park test, that involves a log-gamma GLM regression of var(*ADMINCOST*) on ln $\mathrm{ADM}\hat{\mathrm{IN}}\mathrm{COST}$. The coefficient on $\ln\mathrm{ADM}\hat{\mathrm{IN}}\mathrm{COST}\;$ approximates $\hat{\delta }$.

Notwithstanding, GLM is known to suffer prediction losses if one has heavy tailed data (kurtosis) even after log retransformation of the dependent variable. GLM prediction losses (relative to the log-OLS estimator) increases with the coefficient of kurtosis of the log scale error or when the true underlying model is a log normal with constant error variance (on the log scale). For this reason, Manning and Mullahy [11] and Manning et al.[15] suggest, before using GLM, to assess the form of the log-scale residuals of the OLS regression. If the log-scale residuals are heavy-tailed: leptokurtotic (coefficient of kurtosis > 3) or the log-scale error variance (which increases with skewness of the dependent variable) is greater than or equal to one, then log-OLS regression (with the appropriate retransformation for heteroskedastic variance) may be preferable to GLM. If, however, the log-scale residuals are both leptokurtotic and heteroskedastic, then the results from both log-OLS regression and GLM should be reported and compared. If the probability density function (pdf) of the raw-scale residuals from one of the GLM estimators with a log link are not bell-shaped or skewed bell-shaped, then log-OLS models may be less precise. If the pdf of the raw-scale residuals from GLM are monotonically declining then the appropriate family distribution should be identified using a modified Park test.

Still another problem with GLM is that independent specification of the link and variance functions could lead to bias and estimation inefficiency. Whilst the appropriate variance function may be identified from the modified Park test, one obtains different regression coefficients and inferences on incremental/marginal effects as the link function selected varies. We therefore consider an extended estimating equations (EEE) version of GLM (also referred to as power-GLM or PGLM) that doesn't require *a priori* specification of the link and variance functions. The PGLM/EEE estimator utilizes the following Box-Cox transformation for the link function:

$$X\beta_{k}=\left\{\begin{array}{c} \left({\mathrm{ADM}\hat{\mathrm{IN}}\mathrm{COST}}^{\lambda }-1\right)/\lambda ,\mathrm{if}\;\lambda \neq 0\\ \ln(\mathrm{ADM}\hat{\mathrm{IN}}\mathrm{COST}),\;\mathrm{if}\;\lambda =0 \end{array}\right.$$

Two broad family distributions can be specified: (1) a "power variance" family characterised by: var $\left(\mathrm{ADMINCOST};\theta_{1},\theta_{2}\right)=\theta_{1}{\left(\mathrm{ADM}\hat{\mathrm{IN}}\mathrm{COST}\right)}^{\theta_{2}}$ and (2) a "quadratic variance" family characterised by: var $\left(\mathrm{ADMINCOST};\theta_{1},\theta_{2}\right)=\theta_{1}(\mathrm{ADM}\hat{\mathrm{IN}}\mathrm{COST})+\theta_{2}{\left(\mathrm{ADM}\hat{\mathrm{IN}}\mathrm{COST}\right)}^{2}$, where *θ*_1_, *θ*_2_ together index the appropriate variance distribution for the dataset analysed. By *simultaneous* specification of the link and variance functions, and *joint* estimation of the parameters above, PGLM/EEE is a more flexible and robust estimator especially when no specific link or variance function can be identified. For example, if the GLM over-dispersion parameter $\hat{\delta }$ is a non-integer, then choosing the closest family distribution could lead to efficiency losses [16],[17].

All our analyses were conducted in Microsoft Excel and STATA v. 11.

## Results

### Simulations

**Figure 2:**
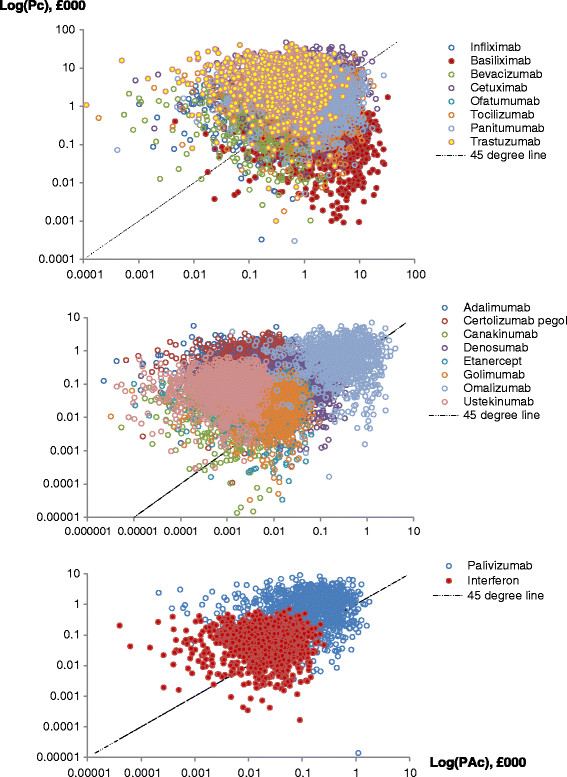
**Simulation outputs.**

Figure 2 below shows the log-log plot of Pc versus PAc from outputs of the simulations for performed for our product sample. The simulations confirm our expectations that the costs of administering biologics subcutaneously or intramuscularly are mainly from the costs incurred before or after physical administration of a drug – although some deviations (inconsistencies) are evident. Observe that for biologics administered subcutaneously or intramuscularly, most of the simulations lie above the 45° line, which equates Pc to PAc. In contrast, the simulations for intravenous biologics fall on either side of the 45° line with the exception of two drugs: trastuzumab and basiliximab. For trastuzumab, most of the simulations fall above the 45° line, which suggests that the associated proximal cost of administering this drug is higher (relative to the physical administration costs). In the case of basiliximab, most the simulations fall below the 45° line indicating that physical administration costs are higher for that drug.

Although Figure 2 provides an idea of the general location of the simulated values for Pc and PAc for each product category, the overlap of data points makes it difficult to tell the pattern for each biologic product. The exact percentage of simulations with the ratio PAc/Pc ≥ 1 can, however, be easily computed from the synthesized data. For the intravenous products, the percentage of simulations with the ratio PAc/Pc ≥ 1 is as follows: basiliximab (95%), bevacizumab (35%), cetuximab (28%), infliximab (28%), oftamumab (49%), panitumumab (46%), tocilizumab (49%) and trastuzumab (9%). For the subcutaneous products, this is as follows: adalimumab (2%), canakinumab (7%), certolizumab pegol (1%), denosumab (22%), etanercept (20%), golimumab (22%), omalizaumab (33%) and ustekinumab (1%). For the intramuscular biologics, we have 17% for palivizumab and 25% for interferon beta-1a. In general, we can say that a higher proportion of the administration costs of biologic drugs given subcutaneously or intramuscularly come from the proximal costs incurred before or after drug administration while for intravenous products, costs are incurred in both cost centres.

**Figure 3:**
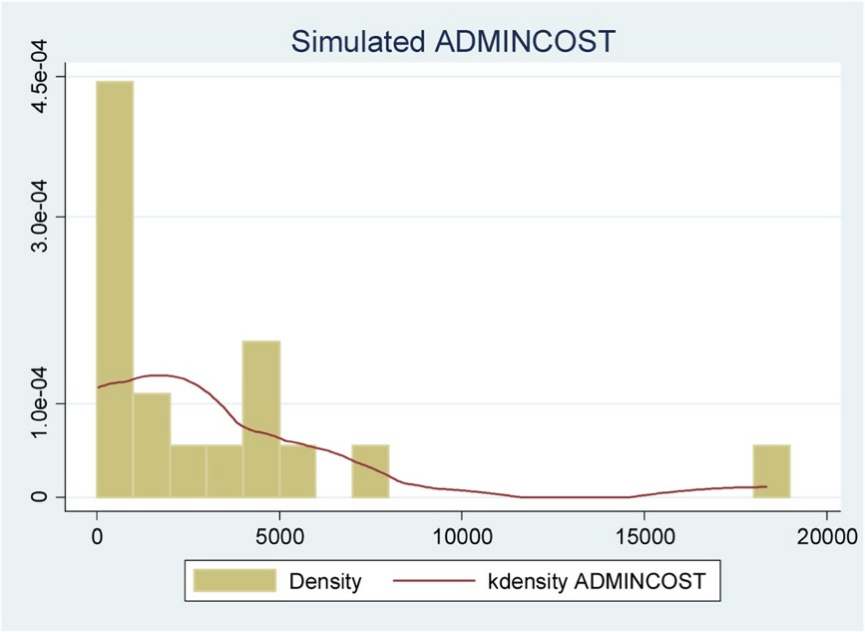
**Distribution of average simulated ADMINCOST.**

An aggregated picture of the simulation outputs above is presented below in the histogram with a kernel density overlay (Figure 3). This shows the empirical distribution of ADMINCOST values averaged over the 1000 simulations for each of the 18 sample products. It is right skewed (coefficient of skewness = 2.3568) with heavy tails (coefficient of kurtosis = 8.6048) – thus confirming the overconcentration of gamma-distributed values. The minimum and maximum values are £35.58 and £18,348.85 respectively; and as often observed of skewed and kurtotic data, the median ADMINCOST (£1414.79) is less than half the mean (£3075.77) and the *standard deviation* (£4477.94) is greater than the mean.

**Figure 4:**
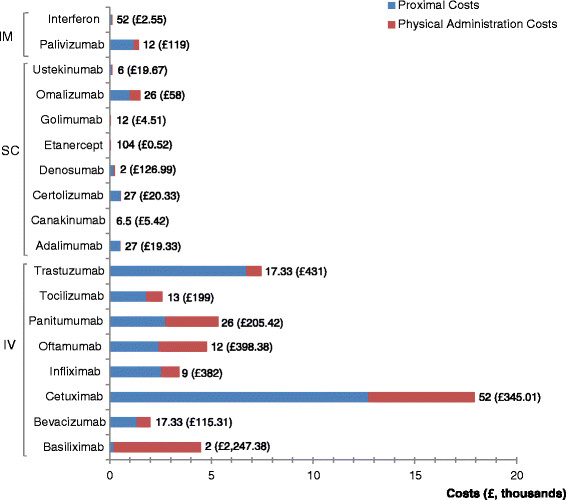
**Variation in drug administration costs.** Notes: IM = intramuscular administration; SC = subcutaneous administration; IV = intravenous administration; values on top of each stacked bar represents DOSFREQ: the number of unit administrations per year; the values in brackets and pound currency refer to the cost per unit administration.

A different picture of the distribution of simulated ADMINCOST values is shown in Figure 4 above. This confirms our prior expectations that the costs of administering biologics given subcutaneously or intramuscularly are lower than that of biologics given intravenously.

However, the numbers on top of each stacked bar in Figure 4 suggests that one has to be cautious when using cost per unit administration to illustrate variations in biologic drug administration costs. Tetteh and Morris[3], for instance, using a selected set of studies offering a comparable scale of measurement for cost per unit administration, report that the administration costs of intravenous biologics appear to be six to eight times that of biologics given subcutaneously or intramuscularly. Differences observed (on the basis of cost per unit administration) disappear, are attenuated or reversed when one considers drug administration costs over a defined period of time. It seems to us that differences in dosing frequency is, at least, one of the reasons why cost per unit administration may not be an appropriate indicator of the variation in administration costs. This unfortunately still leaves unanswered the question: by how much do administration costs differ between biologics given intravenously, subcutaneously or intramuscularly?

### Administration cost algorithm

To answer the question above, we turn to the results of our regression-based algorithm shown in Table 2. Note that the results are for ln $\mathrm{ADM}\hat{\mathrm{IN}}\mathrm{COST}$, not $\mathrm{ADM}\hat{\mathrm{IN}}\mathrm{COST}$. The regression coefficients, nevertheless, carry the desired information as to the link between biologic administration costs and the frequency or route of drug administration. As mentioned earlier, we estimate four nested models A, B, C and D with C as the full unrestricted model. From Table 2, model C offers the best fit with our simulated dataset, having the highest adjusted R^2^, lowest sum of squared residuals and the lowest prediction variance, i.e., the square of SE(ln $\mathrm{ADM}\hat{\mathrm{IN}}\mathrm{COST}$). To ensure robustness, we explore alternative estimators for model C; the results of which are shown in Table 3 below.

**Table 2:** **Identifying the algorithm**

Dependent variable: lnADMINCOST				
	Model A	Model B	Model C	Model D
**Independent variables**	$\hat{\beta_{k}}$ (SE)	$\hat{\beta_{k}}$ (SE)	$\hat{\beta_{k}}$ (SE)	$\hat{\beta_{k}}$ (SE)
SUBCUTANEOUS	-3.2858 (0.57)^***^	-2.6161 (1.106)^**^	-3.2073 (0.902)^***^	—
INTRAMUSCULAR	-2.4097 (0.90)^**^	-2.2784 (1.219)^*^	-5.1856 (1.335)^***^	—
DOSFREQ	-0.0046 (0.011)	-0.0025 (0.012)	0.4206 (0.18)^**^	-0.1022 (0.179)
PRODUCTBUND	—	-0.7395 (1.129)	0.4133 (0.928)	-2.7416 (0.678)^***^
INDICATN	—	-1.0527 (1.219)	-0.2084 (1.467)	-3.7195 (1.693)^**^
DOSFREQ2	—	—	-0.00105 (0.0003)^***^	-0.00075 (0.0004)^*^
DOSFREQ.INDICATN	—	—	-0.3126 (0.172)^*^	0.17 (0.183)
Intercept	8.6223 (0.45)^***^	9.6888 (1.274)^***^	7.1058 (1.521)^***^	11.305 (1.685)^***^
**Regression statistics**				
R^2^ (Adjusted R^2^)	0.7188 (0.6586)	0.7376 (0.627)	0.8881 (0.8098)	0.6957 (0.5689)
F-statistic (>c)	11.9313 (>3.1122)	6.7151 (>2.9961)	11.3369 (>3.0717)	5.4864 (>2.9961)
SSR (N)	17.8023 (18)	16.6714 (18)	7.0858 (18)	19.2688 (18)
SE($\ln\mathrm{ADM}\hat{\mathrm{IN}}\mathrm{COST}$)	1.1276	1.1787	0.8418	1.2672
F-*ratio* (>c)	3.781 (<3.478)	6.764 (>4.1028)	—	10.3161 (>4.1028)

Notes: ${\hat{\beta }}_{k}$ = regression coefficients; ***p < 0.01; **p < 0.05; *p < 0.10; SE = default standard error; F-statistic = F-test for overall significance of the regression; c = critical value for F-test; SSR = residual sum of squares; N = number of observations; F-*ratio* = F-test for comparison with model C; c = critical value for the F-test; SUBCUTANEOUS = dummy variable for subcutaneous delivery; INTRAMUSCULAR = dummy variable for intramuscular delivery; DOSFREQ = dosing frequency; PRODUCTBUND = dummy indicating whether the drug product is sold as a bundled product with some of the equipment and consumables used in drug administration; INDICATN = dummy for type of illness a product is clinically indicated for.

**Table 3:** **Alternative estimators for model C**

Dependent variable: ADMINCOST or lnADMINCOST				
	GLM (log, Gamma)	PGLM/EEE (QV)	GLM (identity Gaussian)	NLLS
**Independent variables**	$\hat{\beta_{k}}$ (SE)	$\hat{\beta_{k}}$ (SE)	$\hat{\beta_{k}}$ (SE)	$\hat{\beta_{k}}$ (SE)
SUBCUTANEOUS	-2.8255 (0.314)^***^	-2.6618 (0.369)^***^	-3.2026 (0.363)^***^	-3.2026 (0.474)^***^
INTRAMUSCULAR	-5.0158 (0.393)^***^	-4.7495 (0.609)^***^	-5.2737 (0.509)^***^	-5.2737 (0.664)^***^
DOSFREQ	0.4015 (0.042)^***^	0.3754 (0.059)^***^	0.428 (0.054)^***^	0.428 (0.07)^***^
PRODUCTBUND	0.2364 (0.198)	0.2336 (0.19)	0.404 (0.269)	0.404 (0.35)
INDICATN	-0.2352 (0.145)	-0.2592 (0.133)^*^	-0.2896 (0.183)	-0.2896 (0.283)
DOSFREQ2	-0.00102 (0.0002)^***^	-0.00093 (0.0001)^***^	-0.00106 (0.0002)^***^	-0.00106 (0.0003)^***^
DOSFREQ.INDICATN	-0.3004 (0.021)^***^	-0.2775 (0.053)^***^	-0.3173 (0.028)^***^	-0.3173 (0.036)^***^
Intercept	7.3703 (0.257)^***^	-0.6008 (0.264)	7.1499 (0.349)^***^	7.1499 (0.455)^***^
**Regression statistics**				
Log pseudo-likelihood (AIC)	-143.3921 (16.377)	—	-16.8641 (2.3182)	—
OD parameter: $\hat{\delta }\left(95\%\;\mathrm{CI}\right)$	1.589 (1.251, 1.921)	—	—	—
Link parameter: $\hat{\lambda }\left(95\%\;\mathrm{CI}\right)$	—	0.078 (-0.2361, 0.3921)	—	—
VF parameter: $\hat{\theta_{1}}\left(95\%\;\mathrm{CI}\right)$	—	0.0304 (-0.0038, 0.0646)	—	—
VF parameter: $\hat{\theta_{2}}\left(95\%\;\mathrm{CI}\right)$	—	0.0899 (-0.0038, 0.1836)	—	—
R^2^ (Adjusted R^2^)	—	—	0.8923 (0.8285)	0.8923 (0.8692)

Notes: ${\hat{\beta }}_{k}$ = regression coefficients; ***p < 0.01; **p < 0.05; *p < 0.10; SE = heteroskedastic-robust standard error; CI = confidence interval; SUBCUTANEOUS = dummy variable for subcutaneous delivery; GLM = generalized linear modelling; PGLM = Power-GLM; EEE = extended estimating equations; QV = quadratic variance function; NLLS = non-linear least squares; INTRAMUSCULAR = dummy variable for intramuscular delivery; DOSFREQ = dosing frequency; PRODUCTBUND = dummy indicating whether the drug product is sold as a bundled product with some of the equipment and consumables used in drug administration; INDICATN = dummy for type of illness a product is clinically indicated for; AIC = Akaike Information Criterion; OD = over-dispersion; VF = variance function.

We found that the raw-scale residuals from a GLM with log-link and gamma family distribution did not exhibit a monotonically declining pdf albeit the residuals were kurtotic (coefficient of kurtosis = 4.3907). The residuals from a standard OLS regression on ADMINCOST were also leptokurtotic (coefficient of kurtosis = 5.080), and remained kurtotic even after log transformation of ADMINCOST (coefficient of kurtosis = 3.1648). Whilst tests for normality of the OLS residuals indicate non-normal data, we could not reject the null of normal data for the residuals from the log-OLS regression (at the 5% significance level). Variants of the Breusch-Pagan test for heteroskedasticity of the log-OLS residuals showed that, at the 5% significance level, we cannot reject the null of constant variance (lowest *p* value = 0.078). Further, a standard Park test, i.e., an auxiliary regression of the form exp $(\hat{{∊}_{i})}=\rho \left(X\right)$ indicated that none of the explanatory variables are statistically significant predictors of log-scale residuals. The log-error variance (i.e., the mean squared error) of the log-OLS model C, however, was less than one – see Table 2. As noted by Manning & Mullahy [11] and Manning et al. [15], these statistics affect the choice between log-OLS and GLM estimators.

A modified Park test following the log-gamma GLM suggests that we cannot reject a Gaussian, poisson, gamma or inverse Gaussian distribution for the GLM variance function. Besides not being able to identify the appropriate GLM link and variance functions, we also know GLM suffers precision losses in the face of heavy-tailed residuals. In fact, a joint estimation of the link and variance functions by PGLM/EEE (QV) rejected a log-gamma GLM^b^. Whilst the regression coefficients from the log-gamma GLM and the PGLM/EEE (QV) are not entirely consistent, the initial coefficient values used in the PGLM/EEE are derived from a gamma GLM with a log-link. Based on the estimated values for $\hat{\lambda }$ and $\hat{\theta_{2}}$, the PGLM/EEE (QV) model identifies NLLS or a log-linear model with Gaussian variance function as the best model fit to our synthesized dataset. We implement the latter using the GLM equivalent of a log-OLS regression as we found that GLM with log-link and Gaussian variance function failed Pregibon's link test. We conjecture that this is because the natural (canonical) link function for a Gaussian family distribution, especially with small samples, is an identity one – not to mention the precision losses from GLM in the face of heavy-tailed residuals.

Note, however, that the log-gamma GLM, PGLM/EEE (QV) and identity-Gaussian GLM models listed in Table 3 all passed the following goodness-of-fit tests: Pearson's test for correlation between raw-scale predictions and residual errors, Pregibon's link test for functional-form specification; and modified Hosmer-Lemeshow tests for systematic patterns in the error residuals. Considering the (borderline) statistically-insignificant tests for skewness, kurtosis and heteroskedasticity; the consistent coefficients from NLLS and the GLM equivalent of log-OLS (plus the observation that none of the explanatory variables in model C are statistically-significant predictors of heteroskedastic variance if it exists), we believe that the underlying true model is closer to a log-normal with homoscedastic variance on the log-scale. We focus therefore our attention on columns 3 and 4 of Table 3: the log pseudo-likelihood and AIC indicates that an identity-Gaussian GLM for lnADMINCOST is a better model fit than a log-gamma GLM for ADMINCOST.

But before proceeding, it is important first to remind ourselves that the reference category is biologic drugs formulated for intravenous administration. The intercept from model C regression represents lnADMINCOST for an intravenous biologic drug indicated for an acute illness and not sold as a bundled product. This, however, carries no particularly meaningful information as it requires DOSFREQ to be zero. There is no point manufacturing and formulating a biologic drug if it is not going to be used. Second, the regression coefficients are semi-elasticities: they represent the *ceteris paribus* percentage change in $\mathrm{ADM}\hat{\mathrm{IN}}\mathrm{COST}$ given a unit change in the explanatory variables [18],[19],[12]. Wooldridge [12] suggest that for continuous variables, the marginal effects $\left(\mathrm{percentage}\;\mathrm{changed}\;\mathrm{in}\mathrm{ADM}\hat{\mathrm{IN}}\mathrm{COST}\right)=100•\hat{\beta_{k}}$ for a unit change in the explanatory variable *X*. For dummy variables, the incremental effects $\left(\mathrm{percentage}\;\mathrm{changed}\;\mathrm{in}\;\mathrm{ADM}\hat{\mathrm{IN}}\mathrm{COST}\right)=100•\left[\exp\left({\hat{\beta }}_{k}\right)-1\right]$. In this section, we follow Kennedy's [18] argument that for dummy variables in a log-OLS regression, the incremental effect is given by: 100•[exp{($\hat{\beta_{k}}$) – 0.5(SE($\hat{\beta_{k}}$)^2^} – 1]. This corrects for small sample bias.

From Table 3, the coefficient for SUBCUTANEOUS has the expected sign and it is statistically significant. This suggests that formulating a biologic drug candidate for subcutaneous rather than intravenous delivery, holding all else constant, reduces $\mathrm{ADM}\hat{\mathrm{IN}}\mathrm{COST}$ by approximately 96.19% (=100•[exp(-3.2026 – .5(.363^2^)) – 1]). The coefficient for INTRAMUSCULAR also has the expected sign and it is statistically significant. This suggests that formulating a biologic drug candidate for intramuscular rather than intravenous delivery will reduce $\mathrm{ADM}\hat{\mathrm{IN}}\mathrm{COST}$ by approximately 99.55% (=100•[exp(-5.2737 – .5(.509^2^)) – 1]) holding all else constant. These percentages do not mean that intravenous products account for over 99% of the variation in administration costs for our product sample – it only refers to isolated incremental effects of the administration-route variables. Also that the difference in the ceteris paribus effects of SUBCUTANEOUS and INTRAMUSCULAR ($\hat{\beta_{0}}-\hat{\beta_{1}}$) is statistically significant (*p* value = 0.0035). That is, there is `strong' evidence of a reduction in ADMINCOST in switching from subcutaneous to intramuscular formulation at the 5% significance level. Note that in models A and B (Table 2), the coefficients for the administration-route variables have the expected signs; they are of the same order of magnitude as the coefficients for model C but are not always statistically significant at the 5% level. We consider that the administration-route variables explain a lot of the variation in lnADMINCOST and ADMINCOST.

The coefficient for the DOSFREQ has the expected sign and it is statistically significant but it should not be interpreted in isolation given the quadratic term DOSFREQ^2^ and the interaction term DOSFREQ.INDICATN. The percentage change in $\mathrm{ADM}\hat{\mathrm{IN}}\mathrm{COST}$ with a unit change in DOSFREQ is not 42.8% (=100•.428). The negative coefficient on DOSFREQ^2^ suggests that the marginal effect of DOSFREQ on ln $\mathrm{ADM}\hat{\mathrm{IN}}\mathrm{COST}$ diminishes with increases in dosing frequency. This curvilinear relationship is as follows:

$$\partial \left(\mathrm{ADM},\hat{\mathrm{IN}},\mathrm{COST}\right)/\partial \mathrm{DOSFREQ}=\;\hat{\beta_{2}}-2\hat{\beta_{5}}\mathrm{DOSFREQ}-\;\hat{\beta_{6}}\mathrm{INDICATN}$$

For an acute illness, INDICATN = 0 and the percentage change in ADMINCOST from moving from once a year dosing to twice a year is 42.59% (=100•[.428 – 2(.00106)(1)]). But moving from 100 to 101 unit administrations per year will only increase $\mathrm{ADM}\hat{\mathrm{IN}}\mathrm{COST}$ by 21.6% (=100•[.428 – 2(.00106)(100)]). For a chronic illness (INDICATN = 1), the corresponding percentage change in $\mathrm{ADM}\hat{\mathrm{IN}}\mathrm{COST}$ of moving from one to two unit administrations per year is 10.86% and -10.13% for moving from 100 to 101 unit administrations per year. The latter result reflects the curvilinear relationship between ADMINCOST and DOSFREQ. Although the coefficient on DOSFREQ suggests that increases in dosing frequency should lead to increases in $\mathrm{ADM}\hat{\mathrm{IN}}\mathrm{COST}$, the coefficient on DOSFREQ^2^ means that beyond some positive value of DOSFREQ (a turning point), increases in dosing frequency will be associated with lower drug administration costs.

For biologics indicated for acute illnesses, this turning point is achieved when DOSREQ is approximately 202 (= .428 divided by 2[.00106]) unit administrations per year. This is roughly a four times a week dosing regimen with perhaps a tapering off of dosing frequency due to improved treatment response or concerns about adverse events. For the two biologic drugs indicated for acute illness, their unit administrations per year are far from 202 so we can safely ignore what happens after the turning point. For biologics indicated for chronic illnesses, this turning point is reached when DOSFREQ is just over 52 (= [.428 – .3173] divided by 2[.00106]) unit administrations per year, i.e., roughly dosing once weekly. Only one of the chronic biologic drugs within our sample (etanercept) has a dosing frequency that greatly exceeds this turning point. We can therefore ignore the turning point knowing the source of this seemingly counterintuitive observation. Indeed, a test for detecting influential (outlier) observations indicated that only one product (etanercept) had an absolute DFITS value greater than twice the threshold of 1.333. (DFITS is the scaled difference in predicted values of ADMINCOST with and without the *j*^th^ observation, in this case etanercept). However, the curvilinear relationship between ADMINCOST and DOSFREQ raises an interesting scenario that, given the peculiar biophysical characteristics and pharmacokinetic profiles of a biologic drug candidate, it is possible to have lower ADMINCOST when DOSFREQ is higher relative to some appropriately defined comparator drug.

For the PRODUCTBUND variable, we found that whilst it did not reach statistical significance, the regression coefficient has the unexpected sign. The size of the coefficient suggests that, holding all else equal, bundling a biologic product with some of the equipment and consumables used in drug administration increases $\mathrm{ADM}\hat{\mathrm{IN}}\mathrm{COST}$ by approximately 44.46% (=100•[exp(.404 – .5(.2693^2^)) – 1]). Since we cannot reject the possibility that the coefficient for PRODUCTBUND is zero, we sought to explore the dataset to see why we observe this result. It turns out that the there is a non-trivial correlation between PRODUCTBUND and SUBCUTANEOUS (correlation coefficient of .6325) and with INTRAMUSCULAR (correlation coefficient of .25). Dropping the administration-route variables in model D (Table 2) produced the expected sign for PRODUCTBUND and it achieved statistical significance. This suggests that bundling a biologic product with some of the equipment and consumables used for drug administration reduces $\mathrm{ADM}\hat{\mathrm{IN}}\mathrm{COST}$ by roughly 95% (=100•[exp(-2.7416 – .5(.678)^2^) – 1]). The effect is similar to that of the administration-route variables. This makes sense as all the products in our sample administered subcutaneously or intramuscularly are, by definition, bundled with some of the equipment and consumables used for administering drugs. Model D, however, suffers from dropping the administration-route variables: its adjusted R^2^ is the lowest (.5689).

The variable INDICATN also shows up with the unexpected sign but it doesn't reach statistical significance in models B and C. This result seems counterintuitive considering the persistence of healthcare resource consumption and expenditures associated with chronic illnesses. In model D (Table 2), however, the variable INDICATN reaches statistical significance. Again, we explored our simulated dataset to find reasons why this might be the case. It turns out that INDICATN is negatively correlated with INTRAMUSCULAR (correlation coefficient of -.4375) and positively correlated with SUBCUTANEOUS (correlation coefficient of .3162). We speculate that the net outcome of these opposing effects is the reason why INDICATN has the negative sign. In fact for the subgroup of intramuscular biologics, there is only one product for acute illness and the other for chronic illness. There are no subcutaneous biologics indicated for acute illnesses in our sample. The apparent counterintuitive result simply reflects the nature of the product sample and simulated dataset. It might not necessarily be observed with a different or updated product sample.

Note that although the variables PRODUCTBUND and INDICATN have unexpected signs and individually they may not always reach statistical significance, they are *jointly significant* in the presence of the other explanatory variables in model C. That is to say, a reduced form of the log-OLS model C that excludes these statistically insignificant variables will carry a lot more of *unexplained* variation in ADMINCOST. As indicated by the F-ratios for the comparisons of model A, B and D with model C in Table 2, there is no justification for excluding these statistically-insignificant variables.

### Validation

As discussed in Section Methods, one must proceed with caution when using the algorithm based on the log-OLS model C to predict the administration costs for a biologic drug candidate. Specifically, one needs a smearing factor to minimize prediction errors. We found that Duan's smearing factor for all product categories was 1.204; and the subgroup-specific smearing factors are as follows: 1.0206 for intravenous products, 1.3799 for subcutaneous products and 1.00 for intramuscular products. The smearing factor from Wooldridge's approach was 1.0287. To identify which of the methods of deriving less biased OLS estimates of $\mathrm{ADM}\hat{\mathrm{IN}}\mathrm{COST}$ is best, we estimated the coefficient of determination (i.e., the square of the correlation coefficient) between the smearing estimates and simulated values for ADMINCOST. This was .822675 for the `normal theory estimates', .822382 for estimates derived using the subgroup-specific smearing factors and .822675 for the Wooldridge approach. There is therefore not much to choose from the three alternatives but we prefer the subgroup-specific smearing factors as it allows us to make cost predictions tailored to specific product formulations; and because the residuals from model C exhibit statistically-insignificant heteroskedasticity that is not explained by any of the explanatory variables.

To illustrate how the algorithm can be used to predict administration costs, consider current attempts to reformulate trastuzumab for subcutaneous delivery. This has been made possible by the feasibility of manufacturing highly-concentrated solutions for monoclonal antibodies and co-formulation with an excipient, recombinant human hyaluronidase that dissolves subcutaneous tissues for rapid drug absorption [20]. Let's assume outcome neutrality between subcutaneous and intravenous delivery of trastuzumab, and the same dosing frequency at three-weekly intervals. Recent phase I trials suggest that subcutaneous and intravenous trastuzumab have indeed comparable efficacy and safety profiles [21]. Also subcutaneous trastuzumab will, by definition, be available as a bundled product: it is mostly likely to be sold together with an automated single-use injectable device in place of manual administration with a syringe [20]. We know trastuzumab is indicated for a chronic illness (see Table 1) so the annual ADMINCOST can be predicted as follows:

$$\mathrm{lnADM}\hat{\mathrm{IN}}\mathrm{COS}T_{\mathrm{IV}}=7.1499-3.2026\left(0\right)-5.2737\left(0\right)+\;.428\left(17.33\right)+\;.404\left(0\right)-\;.2896\left(1\right)-\;.00106\left(17.33^{2}\right)-\;.3173\left(17.33\right)\left(1\right)$$

$$\mathrm{ADM}\hat{\mathrm{IN}}\mathrm{COS}T_{\mathrm{IV}}=\exp\left(8.4604\right)•\hat{ф}\left(=1.0792\right)=£5097.99$$

$$\mathrm{lnADM}\hat{\mathrm{IN}}\mathrm{COS}T_{\mathrm{SC}}=7.1499-3.2026\left(1\right)-5.2737\left(0\right)+\;.428\left(17.33\right)+\;.404\left(1\right)-\;.2896\left(1\right)-\;.00106\left(17.33^{2}\right)-\;.3173\left(17.33\right)\left(1\right)$$

$$\mathrm{ADM}\hat{\mathrm{IN}}\mathrm{COS}T_{\mathrm{SC}}=\exp\left(5.6618\right)•\hat{ф}\left(=1.3799\right)=£396.94$$

where 1.0792 and 1.3799 are the subgroup-specific smearing factors for intravenous and subcutaneous products respectively. Assuming the same price per given dose, this represents a healthcare delivery cost saving of, at least, £4700 per patient per year.

These results are consistent with Samanta et al.'s [22] report that a 100% switch of 200 patients in England receiving intravenous trastuzumab to its subcutaneous equivalent will generate time and resource cost savings of £271,000 in the hospital setting; £1,200,000 in the community setting and £1,500,000 if patients self-administer subcutaneous trastuzumab at home. The savings arise mainly from reduction in pharmacy technicians' time and nursing inputs and time spent in the "IV chair", i.e., being hospitalised to receive an intravenous infusion. Our regression-based algorithm provides a conservative estimate of £940,200 if 200 patients fully switch from intravenous to self-administered subcutaneous trastuzumab. Also De Cock et al. [23] report, from a multi-country, multi-centre time and motion study that resources expended in administering intravenous trastuzumab is mainly in the form of reconstitution in the pharmacy and in "infusion initiation" (no changes in number of patients visits, blood sampling and physician consultation were expected from the switch to subcutaneous trastuzumab). Our results are consistent with this finding as we found that the ratio PAc/Pc is greater than or equal to one in only 9% of 1000 simulations for trastuzumab.

## Discussion

The essence of our administration-cost algorithm is best appreciated when one considers the argument by de la Horie [24] that the reason why biologics are 'so expensive" is because of complex and costly manufacturing, and the need for frequent administration of high doses to be effective. This suggests that healthcare delivery costs could be reduced by manufacturing process innovations that reduce the cost of making biologic drugs as well as reformulation steps that reduce the frequency of dosing. Our results suggest that this is possible but then the curvilinear relationship between ADMINCOST and DOSFREQ means it is also possible for some biologic drugs (given their peculiar biophysical characteristics and pharmacokinetic profiles) to have lower administration costs even with a higher annual dosing frequency (relative to some other biologic drug). However, for our product sample, an increase in DOSFREQ in most cases will be associated with an increase in ADMINCOST albeit at a diminishing marginal rate. But we know that the variables SUBCUTANEOUS and INTRAMUSUCLAR are associated with lower administration costs even when DOSFREQ remains unchanged. We could therefore say the key decision factor is with regards to formulating a product for subcutaneous or intramuscular administration relative to intravenous delivery.

In line with the argument by Eisenstein [25], a one-size-fits-all approach of formulating biologics for intravenous delivery needs to be reconsidered. This, however, should not be taken to mean that all biologics should be manufactured for subcutaneous or intramuscular administration. Intravenous administration has other attributes that make it the most appropriate route for administering a drug. This includes the benefit of immediate injection of active drug moieties into the systemic circulation – something that is desired when an immediate treatment or clinical response is needed. Intravenous delivery is also appropriate for products with narrow therapeutic indices as there is less fluctuations of drug levels in blood plasma. Generally speaking, drugs that need `informed' dosage adjustments (based on accurate measurements of some physiological or biochemical metric) in order to ensure the products deliver positive health benefits (net of safety concerns and risks) are best administered intravenously. Intravenous delivery is also appropriate when a drug cannot be absorbed from the gastrointestinal tract or when a drug cannot be injected into the muscle or other body tissues [26]. Putting aside these clinical reasons, there are manufacturing challenges (issues of technological feasibility) that need to be addressed before the potential efficiency (cost) savings can be realized.

Biologics can be difficult to formulate for subcutaneous or intramuscular delivery as this typically involves injection of small volumes of highly concentrated drug solutions through needles with narrow apertures (needles used for subcutaneous often have an aperture of 0.5 inches whilst needles with 1-2 inch gauges are used for intramuscular delivery). The problem is amplified for biologics that need to be given in high doses and/or have limited solubility. Subsequently, attempts to formulate biologic drugs with fragile molecular structures in high concentrations and small volumes could lead to protein aggregation, undesirable viscosity properties and generally `unstable' drugs that do not retain their biological or biophysical properties. A biologic drug that works well when given in high volumes (because of solubility problems for example) as an intravenous infusion may lose its clinical efficacy or product quality when formulated for subcutaneous or intramuscular delivery in small volumes [27],[25]. What is more if the small volumes of subcutaneous or intramuscular biologics needed to be injected results in an increase in DOSFREQ for a biologic product, the net impact will be determined mainly by the opposing effects of SUBCUTANEOUS or INTRAMUSCULAR and DOSFREQ. Note that this effect of DOSFREQ from frequent subcutaneous or intramuscular injections of small volumes is irrespective of whether the biologic product in question has a longer half-life that, all things being equal, should lead to a lower DOSFREQ.

Another reason why subcutaneous or intramuscular formulation might be associated with an increase in DOSFREQ or higher doses is that biologics given intramuscular or subcutaneously will have go through layers of skin or muscle tissues, and in the process they may be rendered ineffective or fail to reach the desired target sites. That is, if *X* doses of a drug are needed for the desired clinical outcome, some allowance (*W*) must be made for the lost or trapped drug doses by administering *X* + *W* doses of the drug. In fact, this problem is the reason behind the conduct of clinical trials on reformulated trastuzumab, rituximab and immunoglobulin G with recombinant hyaluronidase to enhance drug absorption following subcutaneous administration [27]. Assuming the net effect of SUBCUTANEOUS or INTRAMUSUCLAR and DOSFREQ is a reduction in administration costs, this cost saving might be offset by the fact for a given fixed price per dose, a higher DOSFREQ increases the acquisition costs for that biologic drug. Again, depending on the trade-off between acquisition and administration costs, the overall impact might not be a reduction in disease management or total healthcare delivery costs. Likewise, for the same DOSFREQ, if it costs more to make the reformulated product then to maintain the same price-cost margin, this will lead to a higher product price per dose assuming price demand elasticity remains the same. That said, our regression-based algorithm in these situations should help manufacturers quantify the net impact of their (re) formulation choices on drug administration costs. This, together with considerations of expected product prices, will allow them to generate credible estimates of the total healthcare delivery costs of their products and the likelihood that these products will find favourable recommendations from healthcare payers or providers.

It might be argued that our regression-based algorithm is tied to the product sample selected; that different results may be obtained if a different biologic drug sample is used, perhaps one that has a lot more products that are administered intramuscularly. Besides the observation that biologics are rarely formulated for intramuscular administration, that argument is not specific to our case: it is applicable to almost algorithm that has been developed for one purpose or the other. Our regression-based algorithm may not yield the desired predictions in all situations. That aside, there are non-monetary aspects of drug administration that we haven't considered here; for example, needle phobia and patient discomfort; inconvenience, disruption of daily activities (from more frequent drug dosing) and non-compliance issues that might negatively affect patients' health. We will argue that it is even possible to have an expanded algorithm that when used to predict the impact on drug formulation choice on healthcare delivery considers both the monetary and non-monetary aspects of drug administration. We suggest that further research is undertaken to evaluate, if possible in monetary terms, the non-monetary attributes of drug administration.

Even then an unanswered question is whether biopharmaceutical manufacturers are faced with adequate incentives to consider alternative drug delivery systems or alter their formulation choices as early as possible. From the perspective of the rational or responsible private biopharmaceutical manufacturer, developing alternative drug delivery systems is worth the time, effort and money if the net present value of that decision is positive (see Chess [28]). That is to say, the discounted present value of the stream of incremental quasi-rents (i.e., additional revenue minus the cost of goods) that a reformulated biologic product or an alternative drug delivery system is expected to bring should exceed the discounted present value of the incremental costs of developing the alternative drug delivery system or reformulating a product. Within the UK NHS the use of CEA (HTA) and current efforts to implement value-based pricing (partly based on estimated cost-effectiveness) could and should provide some incentive for manufacturers to consider the relationship between formulation choices and healthcare delivery costs as early as possible in product development but here we cannot say anything about whether this, on its own, will get manufacturers to change their tact. We believe this is also worth considering in future research.

## Conclusions

In this paper, we have evaluated variations in the magnitude of administration costs of biologic drugs, taking care to ensure consistent inclusion of all relevant cost resources. From this, we developed a regression-based algorithm with which manufacturers could possibly predict, during process development, how their choices on manufacturing and formulation may impact on the healthcare delivery costs of their products. Our results confirm the general notion that the administration costs of intravenous products is higher than that or products administered subcutaneously or intramuscularly. We found that formulating a biologic drug for subcutaneous or intramuscular delivery relative to intravenous delivery is associated with lower administration costs that, holding all else equal, should lead to lower total healthcare delivery costs. Increasing the frequency of drug dosing generally will lead to an increase in administration costs but it is possible that this might not always be the case.

There are, however, clinical considerations and manufacturing challenges that might militate against the potential efficiency savings in administration costs from reformulating biologic products or making use of alternative drug delivery systems. But where and when issues of technological feasibility can be dealt with, (bio)pharmaceutical manufacturers could use our algorithm to quantify the net impact on drug administration costs, which together with considerations on the impact on acquisition costs will allow them to generate credible estimates of the total healthcare delivery costs for their products and the likelihood that these products will find favourable recommendations from healthcare payers.

## Endnotes

^a^Our choice of 1000 simulations is not arbitrary. A simulation convergence test, which is not reported in the paper, suggests a virtually flat administration-cost curve with the number of simulation trials ranging from 1000 to 200,000. We do this by running each 1000 simulation 200 times.

^b^In contrast to Basu & Rathouz [16], we found that the PGLM/EEE with a power variance function failed to converge. Misspecification tests for the PGLM/EEE (QV) suggested a good fit with the data: Pearson correlation between the raw-scale residuals and predicted values was not significantly different from zero, and there was no statistically-significant evidence of systematic patterns in the residuals plotted against predicted values.

## Authors' contributions

All authors read and approved the final manuscript.

## Additional files

## Electronic supplementary material

Additional file 1: Appendix A: Dosing regimens modelled. (PDF 52 KB)

Additional file 2: Appendix B: Summary of cost inputs. (PDF 63 KB)

Below are the links to the authors’ original submitted files for images.

Authors’ original file for figure 1

Authors’ original file for figure 2

Authors’ original file for figure 3

Authors’ original file for figure 4
