# Supplementary material for: Evaluating the administration costs of biologic drugs: development of a cost algorithm
Source: Health Econ Rev. 2014 Oct 23;4:26. doi: 10.1186/s13561-014-0026-2 (PMC4883988; doi:10.1186/s13561-014-0026-2)
Supplement: Supplementary file 1 — Additional file 1: Appendix A: Dosing regimens modelled. (PDF 52 KB) [file 13561_2014_26_MOESM1_ESM.pdf]

Appendix A: Dosing regimens modelled

INTRAVENOUS ADMINISTRATION	
**Product name**	**Dosing regimens**
Basiliximab	Standard total dose of 40mg, given in two doses of 20mg each. First dose should be given prior to transplantation surgery, and the second dose given 4 days after transplantation. Reconstituted basiliximab should be administered as an infusion over 20-30 minutes concomitantly with cyclosporine for micro-emulsion or corticosteroid-based immunosuppression (to reduce the risk of organ transplant rejection). We assume 5 mg/kg cyclosporine will be administered per day for two weeks starting the day before transplantation.
Bevacizumab	Recommended dosing is 10mg/kg of body weight given once every two weeks as an intravenous infusion. An initial dose of bevacizumab should be delivered over 90 minutes as an intravenous infusion. If the first infusion is well tolerated, the second infusion may be administered over 60-minutes. If the 60-minute infusion is well tolerated, all subsequent infusions may be administered over 30 minutes. Treatment should be continued until progression of the underlying disease or until acceptable toxicity. Bevacizumab is given *in addition* to platinum-based chemotherapy.
Cetuximab	In all indications, cetuximab must be infused once a week. The initial dose is 400mg cetuximab per m^2 body surface area (BSA) over 2 hours. All subsequent weekly doses are 250mg per m^2 BSA each over 1 hour. Cetuximab should be continued until progression of the underlying disease. Prior to the first infusion, patients must receive premedication with an antihistamine and a corticosteroid to reduce the risk of allergic reactions. Premedication is recommended for all subsequent infusions. Cetuximab can be given as a single agent or as a combination treatment.
Infliximab	Infliximab (3mg/kg) is given concomitantly with methotrexate (MTX) as an intravenous infusion over a 2 hour period. All patients administered are to be observed for at least 1-2 hours post-infusion for acute infusion-related reactions. Patients may be pre-treated with antihistamines, corticosteroids or paracetamol to reduce the risk of infusion-related reactions. This is followed by an additional 3mg/kg infusion doses at 2 and 6 weeks after the first infusion, then every 8 weeks thereafter. We assume 20mg/week of MTX is given with each infusion of infliximab.
Oftamumab	Recommended dose is 300mg ofatumumab for the first infusion and 2000mg for all subsequent infusions. This is given as 8 consecutively weekly infusions followed by a 4-5 week break and later by 4 consecutive monthly (every 4 weeks) infusions. The first and second infusions should be given over 6.5 hours with the 2rd to 12th infusions given over 4 hours. Patients should be pre-medicated (with an analgesic, antihistamine and an intravenous corticosteroid) 30 minutes to 2 hours prior to ofatumumab infusions in an environment where full resuscitation facilities are immediately available. Premedication is to reduce the risk of infusion reactions.

Panitumumab	The recommended dose is 6mg/kg body weight given once every two weeks. Evidence of wild-type KRAS type is required before initiating treatment with panitumumab. The recommended infusion time is approximately 60 minutes. If the first infusion is tolerated, then subsequent infusions may be administered over 30 to 60 minutes. Doses higher than 1000mg should be infused approximately 90 minutes. We assume all infusions are given over 1 hour.
Tocilizumab	Standard dose is 8mg/kg body weight given as an infusion over one hour once every four weeks. Depending on patients' response, dose may be reduced to 4mg/kg body weight, which can then be increased to 8mg/kg body weight when appropriate. Tocilizumab administration requires blood tests before and during treatment to determine if patients have low white blood cell count, platelet count or liver enzyme abnormalities.
Trastuzumab	An initial loading dose of trastuzumab 8mg/kg body weight is given, which is then followed by a maintenance dose of 6mg/kg body weight at three-weekly intervals beginning three weeks after the loading dose. HER2 testing is mandatory prior to initiation of therapy. The loading dose should be administered as a 90-minute infusion by a health professional prepared to manage anaphylaxis and an emergency kit should be available. Patients should be observed for, at least, 6 hours after the start of the first infusion and for two hours after the start of the subsequent infusions for symptoms of fever and chills, and other infusion-related symptoms. All candidates for trastuzumab treatment should undergo baseline cardiac assessment including history and physical examination, electrocardiogram, echocardiogram or MUGA scan or magnetic resonance imaging.
**SUBCUTANEOUS ADMINISTRATION**	
**Product name**	**Dosing regimens**
Adalimumab	The recommended regimen is 80 mg at week 0 followed by 40 mg at week 2. In case there is a need for a more rapid response to therapy, the regimen 160 mg at week 0 (dose can be administered as four injections in one day or as two injections per day for two consecutive days), 80 mg at week 2, can be used with the awareness that the risk for adverse events is higher during induction. After induction treatment, the recommended maintenance dose is 40mg every other week via subcutaneous injection. We consider the 40mg solution for injection in prefilled syringe with needle-guard sold together with one alcohol pad.
Canakinumab	150mg canakinumab administered as a single dose every 8 weeks for CAPS patients with body weight > 40kg or 2mg/kg body weight for CAPS patients with body weight ≥ 15kg and ≤ 40kg. Patients should be monitored for signs and symptoms of infections (including latent or active tuberculosis) before, during and after treatment. It is recommended that neutrophil counts should be assessed prior to initiating treatment, after 1 to 2 months, and periodically thereafter. We consider the injection kit version of canakinumab that contains 1 vial of powder for solution for injection, 1 vial of solvent for injection, 1 injection syringe, 1 safety needle, 2 vial adapters and 4 cleansing swabs.

Certolizumab pegol	Certolizumab pegol (in combination with MTX) is given as a 400mg starting dose (as two injections of 200mg each on one day) at weeks 0, 2 and 4, followed by a maintenance dose of 200mg every two weeks. Certolizumab is formulated as a solution for injection in pre-filled syringes containing 200mg in one ml. Product package consists of two pre-filled syringe to be administered as a subcutaneous injection only and two alcohol-wipes. After proper training, patients may self-inject if their physician determines that it is appropriate and with medical follow-up as necessary. Medical follow-up is needed to check for allergic reactions, opportunistic infections, heart failure, cancer and other disorders.
Denosumab	60 mg of denosumab administered as a single subcutaneous injection once every 6 months into the thigh, abdomen or upper arm. Clinical monitoring of calcium levels for patients predisposed to symptomatic hypocalcaemia supported by adequate intake of calcium and vitamin D supplements before and while on treatment is necessary. Because of the risk of osteonecrosis of the jaw, dental examination with appropriate preventive dentistry should be considered prior to treatment, especially for those with cancer, undergoing chemotherapy or radiotherapy, are taking steroids, do not receive routine dental care or have gum disease. We consider the 60mg solution for injection in pre-filled syringe.
Etanercept	For the treatment of active and progressive psoriatic arthritis, etanercept is administered 25mg twice weekly. We consider the 25mg powder and solvent for solution for injection using a pre-filled syringe. Each pack contains 4 single-dose vials, 4 pre-filled syringes of water for injections and 8 alcohol swabs. Patients should be instructed on self-injection or on giving an injection to a child. A “dose preparation guide” is provided to assist with preparing and giving the injection.
Golimumab	Golimumab is given 50mg once per month, on the same date each month as a subcutaneous injection. Patients may self-inject if properly trained with medical follow-up as necessary. They should be monitored closely for infections including active and inactive (latent) tuberculosis before, during and after treatment. Appropriate screening tests including tuberculin skin or blood test and chest X-ray should be performed in all patients. Patients should be trained by a healthcare professional to prepare the injection and to self-inject. We consider the 50mg solution for injection in a pre-filled syringe.
Omalizumab	The appropriate dose and frequency of Xolair® is determined by baseline IgE (IU/ml) measured before the start of treatment, and on body weight. Prior to administration of the initial dose, patients should have their IgE level determined, and on the basis of these assessments, 75 to 100mg of omalizumab in 1 to 4 injections is given at a time. There are two dosing schedules (based on IgE levels and body weights): every four weeks and every two weeks. The maximum recommended dose is 600mg every two weeks. Here, we modelled the regimen with the maximum recommended dose. We consider 75mg Xolair® solution for injection in a prefilled syringe. Xolair® prefilled syringes are designed to be used by health professionals *only*.

Ustekinumab	Ustekinumab is administered as an initial dose of 45mg subcutaneously followed by a 45mg dose 4 weeks later, and then every 12 weeks thereafter. After proper training in subcutaneous injection technique, patients may self-inject if a physician determines that this is appropriate. Prior to initiating treatments, patients should be evaluated for (latent or inactive) tuberculosis and other infections. Patients receiving ustekinumab should be monitored closely for signs and symptoms of active tuberculosis and other infections during and after treatment. We model the 45mg solution for injection in a pre-filled syringe.
**INTRAMUSCULAR ADMINISTRATION**	
**Product name**	**Dosing regimens**
Pavilizumab	The recommended dose is 15mg/kg body weight given once monthly during anticipated periods of RSV risk. Where possible, the first dose should be administered prior to commencement of the RSV season. Subsequent doses should be administered monthly throughout the RSV season. Medicinal products for the treatment of hypersensitivity reactions including anaphylaxis and anaphylactic shock should be available for immediate use following administration of pavilizumab.
Interferon beta-1a	Recommended dosage for treatment of relapsing multiple sclerosis is 30 micrograms (1 ml solution) given once a week. It is advisable to give, prior to injection and for an additional 24 hours after each injection, an antipyretic analgesic to reduce flu-like symptoms associated with Avonex® administration. Patient with a history of seizures or receiving treatment with anti-epileptics and exhibiting depression should be monitored closely. In addition to laboratory tests required for monitoring patients with multiple sclerosis, complete and differential white blood cell count, platelet counts and blood chemistry including liver function tests is recommended. The drug can be self-administered as long as patients have received adequate training. We focus on the BIO-SET presentation.
