# Supplementary material for: Evaluating the administration costs of biologic drugs: development of a cost algorithm
Source: Health Econ Rev. 2014 Oct 23;4:26. doi: 10.1186/s13561-014-0026-2 (PMC4883988; doi:10.1186/s13561-014-0026-2)
Supplement: Supplementary file 2 — Additional file 2: Appendix B: Summary of cost inputs. (PDF 63 KB) [file 13561_2014_26_MOESM2_ESM.pdf]

Appendix B: Summary of cost inputs

Drug product	Unit costs	Components: Proximal costs	Components: Physical administration costs
**Biologics administered intravenously**			
Basiliximab, Simulect®	Proximal costs:<br>Pretherapy counselling = £78<br>Pharmacy cost = £41	Pretherapy counselling = HRG code PA57Z for "Examination, follow-up, special counselling and other admissions with length of stay one day or more".[1]	Staff costs = half an hour of nurse time (at £40 per hour) in a 24-hour ward (or day ward). Much of this will be attributed to time spent infusing cyclosporin. We allocate this proportionally over the two days on which basiliximab is administered.[2]
	Physical administration costs:<br>Staff costs = £40<br>Concomitant medications = £9.17<br>Equipment and consumables = £20	Pharmacy costs = half an hour of hospital pharmacist's time (at £41 per hour) for reconstituting Simulect® (basiliximab) powder for infusion.[2]	Concomittant medications = 5mL ampoule of ciclosporin (concentration 50mg/mL) = £9.17. Intravenous equivalent of cyclosporin will have to be infused over 2-6 hours. Dosage = 5mg/kg (infused slowly over 6 hours) per day over two weeks starting on the day before transplantation. We assume an average body weight of 70kg.[3,4]<br><br>Equipment and consumables = cost of a number of items: Sterets skin cleansing alcohol swabs = £1.80; (2) Permeable apertured non-woven synthetic adhesive tape BP1988 = £0.90; (3) Nitrile gloves = £0.59 (for a pack of 50); (4) Absorbent gauze tissue BP1988 (500g each) = £7.23; (5) 50mL ampoule of sodium chloride = £4.27. The remainder is assumed to be an allocation for the fixed costs of an infusion pump.[5]
Bevacizumab, Avastin®	Proximal costs:<br>Pretherapy counselling = £78	Pretherapy counselling = HRG code PA57Z for "Examination, follow-up, special counselling and other admissions with length of stay one day or more".[1]	Staff costs = 1.5 hours of nurse time (at £40 per hour) in a 24-hour ward (or day ward) for first infusion. We assume bevacizumab is well-tolerated, meaning second infusion will be given for 1 hour and all subsequent infusions given over 30 minutes.[2]
	Physical administration costs:<br>Staff costs = £40<br>Equipment and consumables = £20	We assume half an hour of hospital pharmacist's time (at £41 per hour) for reconstituting Avastin® (bevacizumab) powder for infusion.[ii]	Equipment and consumables = cost of a number of items: Sterets skin cleansing alcohol swabs = £1.80; (2) Permeable apertured non-woven synthetic adhesive tape BP1988 = £0.90; (3) Nitrile gloves = £0.59 (for a pack of 50); (4) Absorbent gauze tissue BP1988 (500g each) =

			£7.23; (5) 50mL ampoule of sodium chloride = £4.27. The remainder is assumed to be an allocation for the fixed costs of an infusion pump.[iii,v]
Cetuximab, Erbitux®	<u>Proximal costs:</u><br>Pretherapy counselling = £78<br>Pharmacy costs = £41<br>Pretreatment medication costs = £205<br>Progress checks = £40<br>Assessments/ laboratory tests = £208<br><br><u>Physical administration costs:</u><br>Staff cost = £40<br>Equipment and consumables = £20	Pretherapy counselling = HRG code PA57Z for "Examination, follow-up, special counselling and other admissions with length of stay one day or more".[1]<br><br>Pharmacy costs = half an hour of hospital pharmacist's time (at £41 per hour) for reconstituting Erbitux® powder for infusion.[ii]<br><br>Pretreatment medication costs = Cost of precautionary emergency 'equipment' (antihistamines, analgesics, corticosteroids etc.) to reduce risk of infusion-related reactions and hypersensitivity = HRG code WA21W "Other procedures or healthcare problems with critical care". Assume a 50:50 risk of such events occurring.[1]<br><br>Progress checks = 1 hour of nurse time (at £40 per hour) to monitor development of post-infusion acute infusion-related reactions.[2]	Staff costs = 2 hours of nurse time for first infusion (at £40 per hour) and an 1 hour of nurse time for subsequent infusions.[2]<br><br>Equipment and consumables = cost of a number of items: Sterets skin cleansing alcohol swabs = £1.80; (2) Permeable apertured non-woven synthetic adhesive tape BP1988 = £0.90; (3) Nitrile gloves = £0.59 (for a pack of 50); (4) Absorbent gauze tissue BP1988 (500g each) = £7.23; (5) 50mL ampoule of sodium chloride = £4.27. The remainder is assumed to be an allocation for the fixed costs of an infusion pump.[3,5]

		Assessments and laboratory checks = Costs of EGRF tests = £995 for a set of 35 tests. Costs of KRAS tests range from £130-£180 but the manufacturer (Merck Serono) pays for the costs of the tests used in the NHS. We therefore assume zero costs.[6]	
Infliximab, Remicade®	<u>Proximal costs:</u><br>Pretherapy counselling = £78<br>Pharmacy costs = £41<br>Pretreatment medication costs = £205<br>Progress checks = £40<br><br><u>Physical administration costs:</u><br>Staff costs = £40<br>Concomitant medications = £0.36<br>Equipment and consumables = £20	Pretherapy counselling = HRG code PA57Z for "Examination, follow-up, special counselling and other admissions with length of stay one day or more".[1]<br><br>Pharmacy costs = half an hour of hospital pharmacist's time (at £41 per hour) for reconstituting Remicade® (infliximab) powder for infusion.[2]<br><br>Pretreatment medication costs = Cost of precautionary emergency equipment (antihistamines, analgesics, corticosteroids etc.) to reduce risk of infusion-related reactions and hypersensitivity = HRG code WA21W "Other procedures or healthcare problems with critical care". We assume a 50:50 risk of such events occurring.[1]<br><br>Progress checks =2 hours of nurse time (at £40 per hour) to monitor development of post-infusion acute infusion-related reactions.[ii]	Staff costs = 2 hours of nurse time for first infusion (at £40 per hour) and an 1 hour of nurse time for subsequent infusions.[2]<br><br>Concomittant medications = cost of 20mg/week methotrexate. Net price of 100 tablet pack = £35.67.[3]<br><br>Equipment and consumables = cost of a number of items: Sterets skin cleansing alcohol swabs = £1.80; (2) Permeable apertured non-woven synthetic adhesive tape BP1988 = £0.90; (3) Nitrile gloves = £0.59 (for a pack of 50); (4) Absorbent gauze tissue BP1988 (500g each) = £7.23; (5) 50mL ampoule of sodium chloride = £4.27. The remainder is assumed to be an allocation for the fixed costs of an infusion pump.[3,5]
Oftamumab, Arzerra®	<u>Proximal costs:</u><br>Pretherapy counselling = £78<br>Pharmacy costs = £41<br>Pretreatment medication costs = £205<br><br><u>Physical administration costs:</u><br>Staff costs = £40<br>Concomitant medications = £0.36<br>Equipment and consumables = £20	Pretherapy counselling = HRG code PA57Z for "Examination, follow-up, special counselling and other admissions with length of stay one day or more".[1]<br><br>Pharmacy costs = half an hour of hospital pharmacist's time (at £41 per hour) for reconstituting Arzerra® (oftamumab) powder for infusion.[2]	Staff costs = First and second infusions will require 6.5 hours of nurse time (at £40/hour in a 24-hour ward (or day ward). The third and twelfth infusions will be given over 4 hours.[2]<br><br>Concomittant medications = cost of 20mg/week methotrexate. Net price of 100 tablet pack = £35.67.[3]

		Pretreatment medication costs = Cost of precautionary emergency equipment (antihistamines, analgesics, corticosteroids etc.) to reduce risk of infusion-related reactions and hypersensitivity = HRG code WA21W "Other procedures or healthcare problems with critical care". We assume a 50:50 risk of such events occurring.[1]	Equipment and consumables = cost of a number of items: Sterets skin cleansing alcohol swabs = £1.80; (2) Permeable apertured non-woven synthetic adhesive tape BP1988 = £0.90; (3) Nitrile gloves = £0.59 (for a pack of 50); (4) Absorbent gauze tissue BP1988 (500g each) = £7.23; (5) 50mL ampoule of sodium chloride = £4.27. The remainder is assumed to be an allocation for the fixed costs of an infusion pump.[3,5]
Panitumumab, Vectibix®	<u>Proximal costs:</u><br>Pretherapy counselling = £78<br>Pharmacy costs = £41<br>Assessments and laboratory tests = £180<br><br><u>Physical administration costs:</u><br>Staff costs = £40<br>Equipment and consumables = £20	Pretherapy counselling = HRG code PA57Z for "Examination, follow-up, special counselling and other admissions with length of stay 1 day or more".[1]<br><br>Pharmacy costs = half an hour of hospital pharmacist's time (at £41 per hour) for reconstituting Vectibix (panitumumab) powder for infusion.[2]<br><br>Assessments and laboratory tests = Costs of KRAS tests range from £130-£180 but the manufacturer (Merck Serono) pays for the costs of the tests used in the NHS. We therefore assume zero costs.[6]	Staff costs = 2 hours of nurse time for first infusion (at £40 per hour) and 1 hour of nurse time for subsequent infusions.[2]<br><br>Equipment and consumables = cost of a number of items: Sterets skin cleansing alcohol swabs = £1.80; (2) Permeable apertured non-woven synthetic adhesive tape BP1988 = £0.90; (3) Nitrile gloves = £0.59 (for a pack of 50); (4) Absorbent gauze tissue BP1988 (500g each) = £7.23; (5) 50mL ampoule of sodium chloride = £4.27. The remainder is assumed to be an allocation for the fixed costs of an infusion pump.[3,5]
Tocilizumab, Actemra®	<u>Proximal costs:</u><br>Pretherapy counselling = £78<br>Pharmacy costs = £41<br>Progress checks = £40<br>Assessments/ laboratory tests = £3<br><br><u>Physical administration costs:</u><br>Staff costs = £40<br>Equipment and consumables = £20	Pretherapy counselling = HRG code PA57Z for "Examination, follow-up, special counselling and other admissions with length of stay one day or more".[1]<br><br>Pharmacy costs = half an hour of hospital pharmacist's time (at £41 per hour) for reconstituting Actemra® (tocililizumab)	Staff costs = 2 hours of nurse time for first infusion (at £40 per hour) and an 1 hour of nurse time for subsequent infusions.[2]<br><br>Equipment and consumables = cost of a number of items: Sterets skin cleansing alcohol swabs = £1.80; (2) Permeable apertured non-woven

		powder for infusion.[2]<br><br>Progress checks = 1 hour of nurse time (at £40 per hour) to monitor development of post-infusion acute infusion-related reactions.[ii]<br><br>Assessments and laboratory tests = HRG code DAP823 “Haematology (Excluding anti-coagulant services)”. This is to reflect the fact that tocilizumab requires blood tests, before and during treatment, to determine whether patients have low white blood cell count, low platelet count or high liver enzymes.[6]	synthetic adhesive tape BP1988 = £0.90; (3) Nitrile gloves = £0.59 (for a pack of 50); (4) Absorbent gauze tissue BP1988 (500g each) = £7.23; (5) 50mL ampoule of sodium chloride = £4.27. The remainder is assumed to be an allocation for the fixed costs of an infusion pump.[3,5]
Trastuzumab, Herceptin®	<u>Proximal costs:</u><br>Pretherapy counselling = £78<br>Pharmacy costs = £41<br>Progress checks = £40<br>Assessments/ laboratory tests = £223.14<br><br><u>Physical administration costs:</u><br>Staff costs = £40<br>Equipment and consumables = £20	Pretherapy counselling = HRG code PA57Z for “Examination, follow-up, special counselling and other admissions with length of stay one day or more”.[1]<br><br>Pharmacy costs = half an hour of hospital pharmacist's time (at £41 per hour) for reconstituting Herceptin® (trastuzumab) powder for infusion.[2]<br><br>Progress checks = Post-treatment monitoring by a nurse (at £40 per hour) for, at least, 6 hours after start of first infusion, and for 2 hours after the start of subsequent infusions.[2]<br><br>Assessments and laboratory tests = Cost of	Staff costs = 1.5 hours of nurse time (at £40 per hour) in a 24-hour ward (or day ward) for first infusion. We assume the first infusion is well-tolerated and all subsequent infusions given over 30 minutes.[2]<br><br>Equipment and consumables = cost of a number of items: Sterets skin cleansing alcohol swabs = £1.80; (2) Permeable apertured non-woven synthetic adhesive tape BP1988 = £0.90; (3) Nitrile gloves = £0.59 (for a pack of 50); (4) Absorbent gauze tissue BP1988 (500g each) = £7.23; (5) 50mL ampoule of sodium chloride = £4.27. The remainder is assumed to be an allocation for the fixed costs of an infusion pump.[3,5]

		HER2 testing (= £170 for a FISH test) and assessment of cardio-toxicity: the latter may involve “Simple echocardiogram, 19 years and over” (HRG code RA60A = £79), "Cardiac magnetic resonance imaging" (HRG code RA652 = £449) or “Electrocardiogram monitoring and stress testing” (HRG code EA472 = £112). We assume there is a 1:3 chance of using any of these procedures to assess cardio-toxicity due to trastuzumab.[1]	
**Biologics administered subcutaneously**			
Adalimumab, Humira®	Proximal costs:<br>Education for self-administration = £0.463<br>Pretherapy counselling = £30<br>Assessments/ laboratory tests = £17.5	Training on how to prepare and self-administer adalimumab, assumed to require half an hour of GP nurse time (at £25 per hour of patient-related work); a one-off cost.[2]	Sharpsafe® or Sharpsguard® (disposal container for needles, syringes and vials) and sticking plasters needed after injection. We assume a permeable, apertured non-woven synthetic adhesive tape BP1988 = £0.90 should be adequate for a month; plus a 1-litre Sharpsguard® container (£0.85).[5]
	Physical administration costs:<br>Equipment and consumables = £0.2565	Advice and monitoring of patients after treatment = OPATT code WA20Y “Examination, follow-up or special screening without critical care” (£30); a one-off cost.[1]<br><br>Monitoring for infections (i.e., visits by a community nurse specializing in infections) = Community and outreach nursing services code CN207AF “Band 7 - Infectious Diseases: Adult: Face to face” (£70). We assume these visits are done once a month.[1]	
Canakinumab, Ilaris®	Proximal costs:<br>Education for self-administration = £0.4464<br>Pretherapy counselling = £30	Training on how to prepare and self-administer adalimumab, assumed to require 1/2 hour of GP nurse time (at £25 per hour of patient-related work); a one-off cost.[2]	Sharpsafe® or Sharpsguard® (disposal container for needles, syringes and vials) and sticking plasters needed after injection. We assume a permeable, apertured non-woven synthetic adhesive tape BP1988 = £0.90 should be adequate for a month; plus a 1-litre Sharpsguard® container (£0.85) for needle disposal.[5]
	Physical administration costs:<br>Equipment and consumables = £0.3558	Advice and monitoring of patients after treatment = OPATT code WA20Y	

		"Examination, follow-up or special screening without critical care" (£30); a one-off cost.[1]	
Certolizumab pegol, Cimzia®	<u>Proximal costs:</u><br>Education for self-administration = £0.962<br>Pretherapy counselling = £30<br>Assessments/ laboratory tests = £18.5<br><br><u>Physical administration costs:</u><br>Equipment and consumables = £0.2565	Training on how to prepare and self-administer adalimumab, assumed to require half an hour of GP nurse time (at £25 per hour of patient-related work).[2]<br><br>Advice and monitoring of patients after treatment = OPATT code WA20Y "Examination, follow-up or special screening without critical care" (£30).[1]<br><br>Assessing neutrophil counts and monitoring for infections (i.e., visits by a community nurse specializing in infections) = Direct Access: Pathology Services code DAP823 "Haematology" (£3) + DAP841 "Biochemistry" (£1) + Community and outreach nursing services code CN207AF "Band 7 - Infectious Diseases: Adult: Face to face" (£70). We assume these tests/visits are done once a month.[1]	Sharpsafe® or Sharpsguard® (disposal container for needles, syringes and vials) and sticking plasters needed for administration. We assume a permeable, apertured non-woven synthetic adhesive tape BP1988 = £0.90 should be adequate for a month; plus a 1-litre Sharpsguard® container (£0.85).[5]
Denosumab, Prolia®	<u>Proximal costs:</u><br>Education for self-administration = £12.5<br>Pretherapy counselling = £30<br>Assessments/ laboratory tests = £77.5<br><br><u>Physical administration costs:</u><br>Concomittant medications = £27.365<br>Equipment and consumables = £0.875	Training on how to prepare and self-administer adalimumab, assumed to require half an hour of GP nurse time (at £25 per hour of patient-related work).[2]<br><br>Advice and monitoring of patients of calcium levels after treatment = OPATT code WA20Y "Examination, follow-up or special screening without critical care" (£30). We assume this is done twice a year.[1]<br><br>Dental examination with appropriate preventive dentistry prior to treatment with	Calcium and vitamin D supplements to counter the risk of symptomatic hypocalcemia. We assume daily intake of such supplements. We use the price of pack of 30 chewable tablets of calcium and colecalciferol (Kalipros-D®) = £4.21. We assume 13 packs for an annual supply.[3]<br><br>Sharpsafe® or Sharpsguard® (disposal container for needles, syringes and vials) and sticking plasters needed for administration. We assume permeable apertured non-woven synthetic adhesive tape BP1988 = £0.90 should be adequate for a month; plus a 1-litre Sharpsguard® container (£0.85).[5]

		Prolia® = OPROC code CZ39Y "Intermediate dental procedures, 19 years and over" (£155).[1]	
Etanercept, Enbrel®	Proximal costs:<br>Education for self-administration = £0.2404<br>Pretherapy counselling = £30<br><br>Physical administration costs:<br>Equipment and consumables = £0.112	Training on how to prepare and self-administer adalimumab, assumed to require half an hour of GP nurse time (at £25 per hour of patient-related work).[2]<br><br>Medical check for signs and symptoms of tuberculosis prior to treatment = OPATT code WA20Y “Examination, follow-up or special screening without critical care” (£30).[1]	Sharpsafe® or Sharpsguard® (disposal container for needles, syringes and vials) and sticking plasters needed for administration. We assume a permeable, apertured non-woven synthetic adhesive tape BP1988 = £0.90 should be adequate for a month; plus a 1-litre Sharpsguard® container (£0.85).[5]
Golimumab, Simponi®	Proximal costs:<br>Education for self-administration = £2.0833<br>Pretherapy counselling = £30<br><br>Physical administration costs:<br>Equipment and consumables = £0.9708	Training on how to prepare and self-administer adalimumab, assumed to require half an hour of GP nurse time (at £25 per hour of patient-related work).[2]<br><br>Medical check for signs and symptoms of tuberculosis prior to treatment = OPATT code WA20Y “Examination, follow-up or special screening without critical care” (£30).[1]	Sharpsafe® or Sharpsguard® (disposal container for needles, syringes and vials) and sticking plasters needed for administration. We assume a permeable, apertured non-woven synthetic adhesive tape BP1988 = £0.90 should be adequate for a month; plus a 1-litre Sharpsguard® container (£0.85).[5]
Omalizumab, Xolair®	Proximal costs:<br>Pretherapy counselling = £30<br>Assessments/ laboratory tests = £8<br><br>Physical administration costs:<br>Staff costs = £40	Medical checks for dose determination and “optimization” of other anti-asthma medications prior to treatment = OPATT code WA20Y “Examination, follow-up or special screening without critical care” (£30).[1]<br><br>Assessment of IgE levels prior to treatment = Direct Access: Pathology Services code DAP830 “Immunology” (£8).[1]	Half an hour of nurse time (at £40/hour in a 24-hour ward (or day ward) to administer 4 injections subcutaneously (at a time).[2]
Ustekinumab, Stelara®	Proximal costs:<br>Education for self-administration = £4.1667<br>Pretherapy counselling = £30<br>Assessments/ laboratory tests = £12.33	Training on how to prepare and self-administer adalimumab, assumed to require 1/2 hour of GP nurse time (at £25 per hour of patient-related work).[2]	Sharpsafe® or Sharpsguard® (disposal container for needles, syringes and vials) and sticking plasters needed for administration. We assume a permeable, apertured non-woven synthetic

	<u>Physical administration costs:</u><br>Equipment and consumables = £0.2554	Advice and monitoring of patients after treatment = OPATT code WA20Y "Examination, follow-up or special screening without critical care" (£30).[1]<br><br>Assessing neutrophil counts and monitoring for infections (i.e., visits by a community nurse specializing in infections) = Direct Access: Pathology Services code DAP823 "Haematology" (£3) + DAP841 "Biochemistry" (£1) + Community and outreach nursing services code CN207AF "Band 7 - Infectious Diseases: Adult: Face to face" (£70). We assume the frequency of these tests/visits will follow the drug's administration schedule.[1]	adhesive tape BP1988 = £0.90 should be adequate for a month; plus a 1-litre Sharpsguard® container (£0.85).[5]
**Biologics administered intramuscularly**			
Palivizumab, Synagis®	<u>Proximal costs:</u><br>Pretherapy counselling = £78<br>Pharmacy costs = £41<br>Pretreatment medication costs = £138<br><br><u>Physical administration costs:</u><br>Staff costs = £40	Advice on when to return for additional doses and adjustments for children undergoing cardiac bypass = OPATT code WA20Y "Examination, follow-up or special screening without critical care" (£30).[1]<br><br>Cost of precautionary emergency 'equipment' (antihistamines, analgesics, corticosteroids etc.) to reduce risk of infusion-related reactions and hypersensitivity = OPATT code WA21Y "Other procedures or healthcare problems with critical care" (£138). We assume a 50:50 risk of such events occurring.[1]	Half an hour of nurse time (at £40/hour in a 24-hour ward (or day ward) to administer drug intramuscularly.[2]
Interferon beta-1a, Avonex®	<u>Proximal costs:</u><br>Education for self-administration = £0.48<br>Pretherapy counselling = £30<br>Pretreatment medication costs = £0.057<br>Assessments/ laboratory tests = £1	Training on how to prepare and self-administer Avonex®, assumed to require half an hour of GP nurse time (at £25 per hour of patient-related work).[2]	Alcohol swipes and sticking plasters needed for administration. We assume Sterets skin cleansing alcohol swabs = £1.80, plus a permeable apertured non-woven synthetic adhesive tape BP1988 = £0.90 should be adequate for a month.[5]

	Physical administration costs:<br>Equipment and consumables = £0.68	Advice and monitoring of patients with cardiac disease, exhibiting depression, with a history of seizures or those receiving treatment with anti-epileptics = OPATT code WA20Y "Examination, follow-up or special screening without critical care" (£30).[1]<br><br>Antipyretic analgesic (500 mg paracetamol tablets) to decrease flu-like symptoms associated with Avonex®. It should be given prior to injection and for an additional 24 hours after each injection. We assume a pack of 100 tablets at £2.94 should be enough for a year as symptoms subside over time.[5]<br><br>Monitoring of laboratory abnormalities associated with use of Avonex® covering complete and differential white blood cell count, platelet counts and blood chemistry including liver function tests = Direct Access: Pathology Services code DAP823 "Haematology" (£3)and DAP841 "Biochemistry" (£1). We assume these tests are done once a month.[1]	

[1] NHS Reference Costs 2011-2012.
[2] PSSRU Unit Costs for Health and Social Care 2012.
[3] British National Formulary (BNF) 65 July 2013.
[4] Electronic Medicines Compendium (eMc).
[5] BNF 65, July 2013; Electronic Drug Tariff.
[6] NICE Technology Appraisal Guidance TA118; Mellor, L & Bourne, N (2011) presentation on "KRAS testing procedures" – available at: http://www.google.co.uk/url?sa=t&rct=j&q=&esrc=s&frm=1&source=web&cd=1&ved=0CDYQFjAA&url=http%3A%2F%2Fwww.kentmedwaycancernetwork.nhs.uk%2FEasySiteWeb%2FGatewayLink.aspx%3FalId%3D205490&ei=g8TWUqiYKYiohAeKvoCICA&usg=AFQjCNFCTZ93s5k0QWgXEdeby3Oup89NSQ (Accessed: 12/08/2013)
