# Supplementary figures and images for: Evaluating the administration costs of biologic drugs: development of a cost algorithm
Source: Health Econ Rev. 2014 Oct 23;4:26. doi: 10.1186/s13561-014-0026-2 (PMC4883988; doi:10.1186/s13561-014-0026-2)

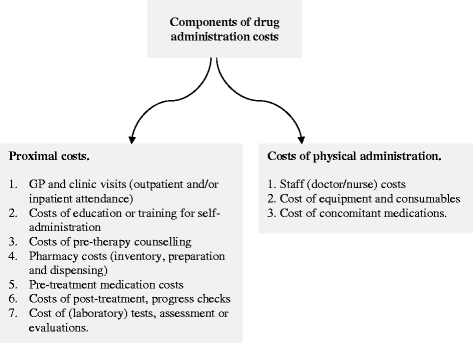

Supplement: Supplementary file 3 — Authors’ original file for figure 1 [file 13561_2014_26_MOESM3_ESM.gif]

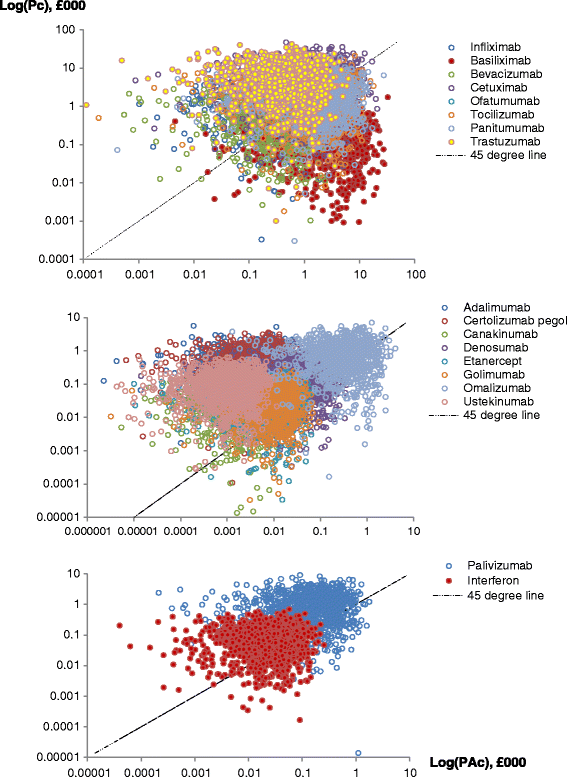

Supplement: Supplementary file 4 — Authors’ original file for figure 2 [file 13561_2014_26_MOESM4_ESM.gif]

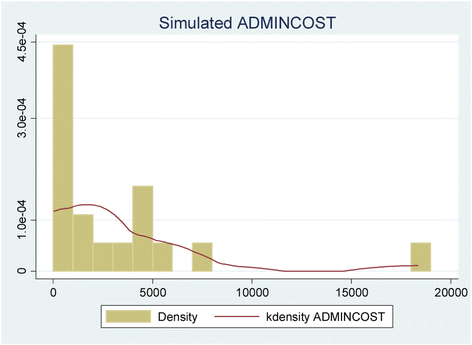

Supplement: Supplementary file 5 — Authors’ original file for figure 3 [file 13561_2014_26_MOESM5_ESM.gif]

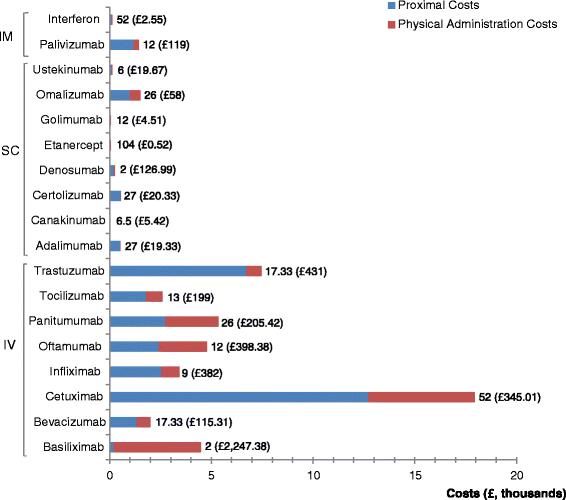

Supplement: Supplementary file 6 — Authors’ original file for figure 4 [file 13561_2014_26_MOESM6_ESM.gif]
